# Beneficial impact of Gpnmb and its significance as a biomarker in nonalcoholic steatohepatitis

**DOI:** 10.1038/srep16920

**Published:** 2015-11-19

**Authors:** Akihiro Katayama, Atsuko Nakatsuka, Jun Eguchi, Kazutoshi Murakami, Sanae Teshigawara, Motoko Kanzaki, Tomokazu Nunoue, Kazuyuki Hida, Nozomu Wada, Tetsuya Yasunaka, Fusao Ikeda, Akinobu Takaki, Kazuhide Yamamoto, Hiroshi Kiyonari, Hirofumi Makino, Jun Wada

**Affiliations:** 1 Department of Medicine and Clinical Science, Okayama University Graduate School of Medicine, Dentistry and Pharmaceutical Sciences, Kita-ku, Okayama 700-8558, Japan; 2 Department of General Medicine, Okayama University Graduate School of Medicine, Dentistry and Pharmaceutical Sciences, Kita-ku, Okayama 700-8558, Japan; 3 Department of Diabetes and Metabolism, National Hospital Organization Okayama Medical center, Kita-ku, Okayama 701-1154, Japan; 4 Department of Gastroenterology and Hepatology, Okayama University Graduate School of Medicine, Dentistry and Pharmaceutical Sciences, Kita-ku, Okayama 700-8558, Japan; 5 Animal Resource Development Unit, 2-2-3 Minatojima Minami, Chuou-ku, Kobe 650-0047, Japan; 6 Genetic Engineering Team, RIKEN Center for Life Science Technologies, 2-2-3 Minatojima Minami, Chuou-ku, Kobe 650-0047, Japan

## Abstract

Nonalcoholic fatty liver disease (NAFLD) is the most common cause of chronic liver disease worldwide. Gpnmb is classified as a type 1 membrane protein and its soluble form is secreted by ADAM10-mediated cleavage. Gpnmb mRNA was found in the Kupffer cells and white adipose tissues (WATs) and its upregulation in obesity was recently found. Here, we generated aP2 promoter-driven Gpnmb transgenic (Tg) mice and the overexpression of Gpnmb ameliorated the fat accumulation and fibrosis of the liver in diet-induced obesity model. Soluble form of Gpnmb in sera was elevated in Gpnmb Tg mice and Gpnmb concentrated in hepatic macrophages and stellate cells interacted with calnexin, which resulted in the reduction of oxidative stress. In the patients with non-alcoholic steatohepatitis, serum soluble GPNMB concentrations were higher compared with the patients with simple steatosis. The GPNMB is a promising biomarker and therapeutic target for the development and progression of NAFLD in obesity.

Nonalcoholic fatty liver disease (NAFLD) is now considered as the most common cause of chronic liver disease worldwide and the prevalence increased beyond 30% in developing countries^1^. NAFLD is consisted of simple steatosis (SS) and nonalcoholic steatohepatitis (NASH), the latter is characterized by presence of hepatic steatosis and inflammation with hepatocyte injury (ballooning) with or without fibrosis, and it can progress to cirrhosis, liver failure and liver cancer^2^. NAFLD is often associated with components of metabolic syndrome such as abdominal obesity, insulin resistance, hypertension, and dyslipidemia, thus metabolic syndrome is a main risk factor for NAFLD^3^. The accumulation of visceral adipose tissues is closely related to release of free fatty acid (FFA), pro-inflammatory cytokines and the dysregulation of secretion of various adipokines such as decrease in adiponectin and elevation of leptin, tumor necrosis factor-α (TNFα), resistin and visceral adipose tissue-derived serine proteinase inhibitor (vaspin)^4^. Since portal blood flow leads to the exposure of FFA and various adipokines to liver tissue, the accumulation of visceral adipose tissues directly influences the development of NAFLD by affecting FFA influx, *de novo* lipid synthesis, oxidative stress and inflammation in the liver^5^. Oxidative stress triggers the immune responses and activates various inflammatory cells such as hapatic macrophages^6^,^7^, which result in liver fibrosis^8^. Although the tight correlation between NAFLD and metabolic syndrome draws the attention, the presence of molecules which connect the pathobiology of the visceral adipose tissues and liver is not totally unknown.

In previous investigation, we attempted to isolate the genes which upregulate in the visceral adipose tissues of Otsuka Long-Evans Tokushima fatty (OLETF) rats, which is an animal model for metabolic syndrome and type 2 diabetes^9^,^10^. During the screening, we isolated the cDNA fragment of Gpnmb (glycoprotein nonmelanoma protein B), which was prominently upregulated in the visceral adipose tissues compared with lean Long-Evans Tokushima Otsuka (LETO) rats (Supplementary Fig. 1a). Gpnmb is classified as a type 1 membrane protein consisting of N-terminal signal peptide, Arg-Gly-Asp (RGD) domain, a polycystic kidney disease-like domain (PKD), and a proline-rich repeat domain (PRRD)^11^,^12^. The soluble form of Gpnmb is secreted from various cells by a disintegrin and metallopeptidase domain 10 (ADAM10)-mediated cleavage^13^,^14^,^15^. Gpnmb was originally discovered as osteoactivin from an animal model of osteopetrosis^16^, the expression is stimulated by bone morphogenetic protein-2 (BMP-2) and homeodomain transcription factors, Dlx3, Dlx5, and Msx2, and it serves as a positive regulator for the osteoblastogenesis^17^,^18^. Independently, Gpnmb was also identified from melanoma cell lines^19^,^20^, breast cancer^21^, dendritic cells (DCs) and T cells as dendritic cell heparin sulfate proteoglycan integrin dependent ligand (DC-HIL)^22^,^23^, and human bone marrow cells as human hematopoietic growth factor inducible neurokinin (HGFIN)^24^. Although the high expression of GPNMB in breast cancer tissues is reported as a poor prognostic indicator for recurrence^21^ and an antibody-drug conjugate targeting GPNMB has been postulated^25^, many researchers are trying to elucidate the role of Gpnmb in immune responses. Gpnmb is induced in macrophages and RAW264.7 cells by interferon-γ (IFN-γ) and lipopolysaccharide, and negatively regulates the production of cytokines IL-6 and IL-12^26^. The binding between antigen presenting dendritic cells and T cells negatively regulated the T cell activation^23^,^27^ and this process is mediated by the interaction between Gpnmb on DCs and syndecan-4 on T cells in allogenic^28^,^29^ and autoimmune^30^ responses.

Here, we generated aP2 promoter-driven Gpnmb transgenic (Tg) and deficient mice carrying C-terminal-truncated form of Gpnmb with 150 amino acids, and they were subjected to the diet-induced obesity model, since we found Gpnmb is highly expressed in white adipose tissues (WATs) in obesity and it has also been reported to be induced in obese adipose tissue macrophages, recently^31^. Initially, we hypothesize that the tissue inflammation in WATs and insulin resistance would be influenced by the genetic manipulation of Gpnmb. Surprisingly, the fat accumulation, inflammation in WATs, and insulin sensitivity were not altered but the overexpression of Gpnmb improved and ameliorated the fat accumulation and tissue fibrosis in the liver. Furthermore, we have found the presence of soluble and secreted form of Gpnmb in rodents and human sera and postulated that it is a promising biomarker for NASH and Gpnmb may be a new therapeutic key molecule for NAFLD.

## Results

### Gpnmb is induced in white adipose tissues (WATs) under obese state and its soluble forms are secreted from cultured adipocytes

**Figure 1:**
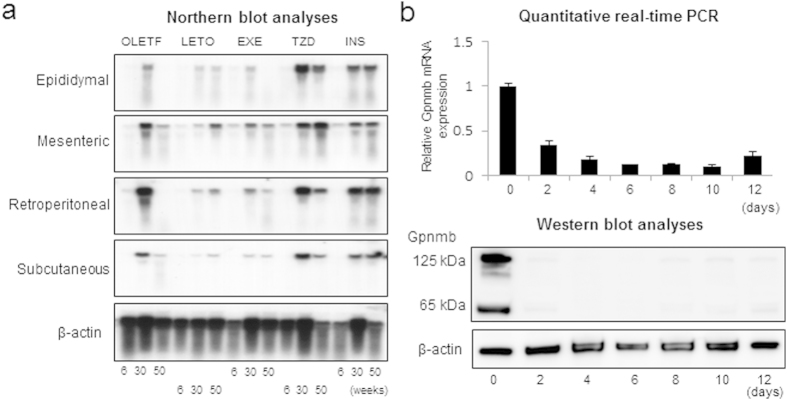
mRNA expression levels of *Gpnmb* in OLETF rats and 3T3-L1 cells. (**a**) Northern blot analyses in visceral and subdermal white adipose tissues in LETO, and OLETF rats with voluntary exercise (EXE), OLETF rats administered with pioglitazone (TZD), and insulin (INS). (**b**) Quantitative real-time PCR of Gpnmb (upper panel) and western blot analyses (lower panel) in 3T3-L1 cells.

We identified Gpnmb mRNA by its upregulation in WATs of OLETF rats in a previous study (Supplementary Fig. 1a)^9^. To investigate the regulation of Gpnmb gene expression in various WATs, OLETF rats were subjected to voluntary exercise (EXE), treatments with pioglitazone (TZD) or insulin (INS), and they were sacrificed at 6, 30 and 50 weeks of age. The body and fat pad weight of OLETF rats peaked at 30 weeks of age, and thereafter they progressively lost their weight for the next 20 weeks along with the rise in HbA1c levels. In TZD and INS groups, OLETF rats progressively gained body and fat pad weight throughout the observation period. Gpnmb mRNA increased with development of obesity until 30 weeks of age, i.e. the peak of body weight in control OLETF rats, and it declined at 50 weeks of age in both subdermal and visceral WATs (Fig. 1a). In EXE group, Gpnmb mRNA expression was reduced to similar levels of lean LETO rats. In contrast, mRNA expression of Gpnmb remained at a high levels until 50 weeks of age in TZD and INS groups (Fig. 1a). The results indicated that the expression of Gpnmb in WATs closely linked to the WAT mass and development of obesity. We next separated the mature adipocytes and stromal vascular fractions (SVF) and found that Gpnmb was mainly expressed in SVF in C57BL/6JJcl mice under HFHS chow (Supplementary Fig. 1b). Recently, Gpnmb has been reported to localize in adipose tissue macrophages (ATMs) and it is a marker for obesity-induced infiltration of ATMs^31^. Then, we further investigated whether Gpnmb is expressed during 3T3-L1 adipocyte differentiation. Gpnmb mRNA was abundantly expressed in preadipocytes and it acutely declined after the induction of adipocyte differentiation (Fig. 1b, upper panel). Since the soluble forms of Gpnmb have been reported in various tumor cell lines, osteoblasts and DCs, we investigated the soluble forms of Gpnmb in supernatant of 3T3-L1 adipocyte culture. Both glycosylated (~125 kDa) and unglycosylated (~65 kDa) secreted forms of Gpnmb were found by Western blot analyses and they again demonstrated the highest expression in preadipocytes (Fig. 1b, lower panels).

### Overexpression of Gpnmb ameliorates liver fibrosis in obesity

**Figure 2:**
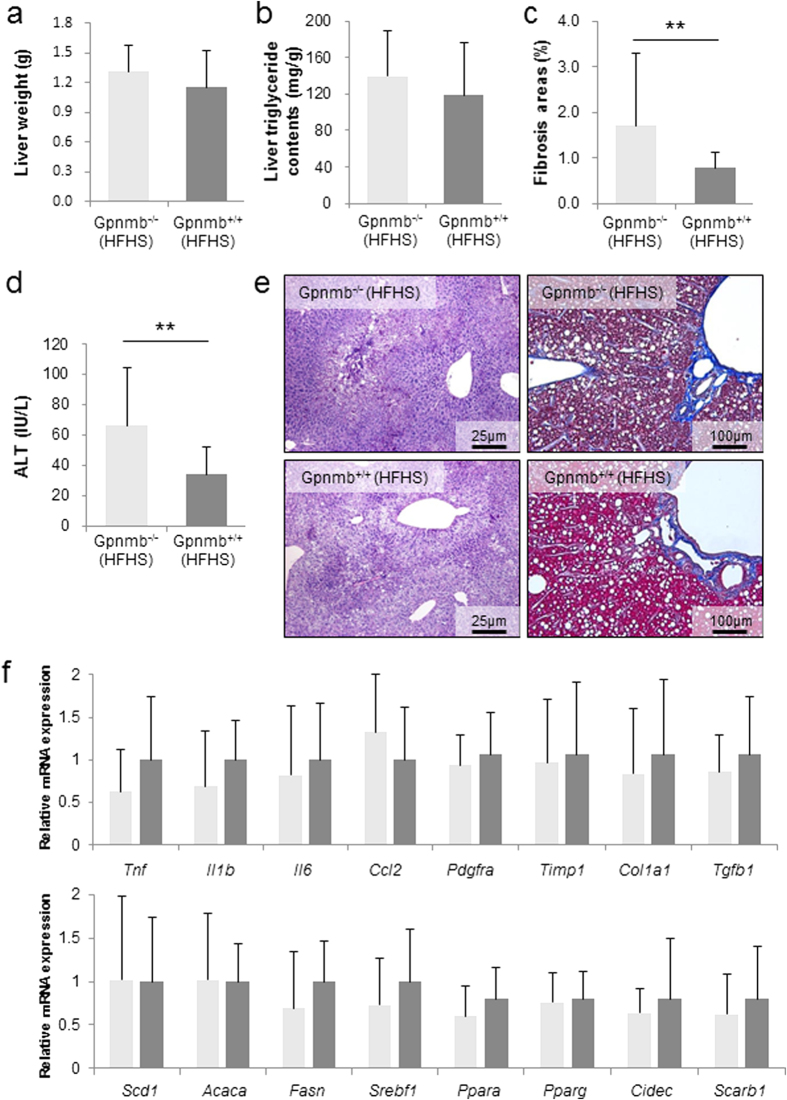
Deterioration of liver fibrosis in Gpnmb^−/−^ mice under HFHS chow. (**a,b**) Liver weight and triglyceride contents of Gpnmb^−/−^ and Gpnmb^+/+^ mice under HFHS chow. (**c,d**) Liver fibrosis areas and serum alanine aminotransferase (ALT) levels of Gpnmb^−/−^ and Gpnmb^+/+^ mice under HFHS chow. (**e**) Periodic acid-Schiff (PAS) staining (left panels) and Azan-Mallory staining (right panels) of liver tissues of Gpnmb^−/−^ and Gpnmb^+/+^ mice under HFHS chow. (**f**) Analyses of inflammation, fibrosis and fatty acid synthesis-related genes. mRNA levels of liver tissues of Gpnmb^−/−^ and Gpnmb^+/+^ mice under HFHS chow by quantitative real-time PCR. All data are presented as mean ± standard deviation (SD) n = 8. **p < 0.01 *v.s.* Gpnmb^+/+^ mice.

Since Gpnmb is predominantly expressed in SVF of WATs in C57BL/6JJcl mice fed with HFHS chow, we generated Gpnmb^−/−^ mice by backcrossing DBA2J mice naturally occurring stop codon mutation of Gpnmb (R150X) to C57BL/6JJcl mice in order to explore the role of Gpnmb in obesity and metabolic syndrome. We confirmed the absence of both glycosylated and unglycosylated forms of Gpnmb in the epididymal WATs by Western blot analyses (Supplementary Fig. 2a). However, under both STD and HFHS chows, the body weight, fat pad weight, adipocyte size, glucose tolerance and insulin sensitivity were not altered in Gpnmb^−/−^ mice (Supplementary Fig. 2b–g). Serum lipid profile in Gpnmb^−/−^ mice demonstrated the significant elevation of total cholesterol and LDL-cholesterol (Supplementary Fig. 3a). The liver weight and triglyceride contents of Gpnmb^−/−^ and Gpnmb^+/+^ mice revealed no differences (Fig. 2a,b). Gpnmb^−/−^ mice demonstrated sparse perihepatocyte fibrosis revealed by Azan-Mallory stain (1.71 ± 1.57 v.s. 0.786 ± 0.335% area, p = 0.001) (Fig. 2c,e) and mild elevation of serum ALT in Gpnmb^−/−^ mice compared with WT mice (66.1 ± 38.2 v.s. 34.1 ± 18.0 U/L, p = 0.002) (Fig. 2d). Sirius Red staining also confirmed that fibrosis areas significantly increased in Gpnmb^−/−^ mice, although liver hydroxyproline contents were not altered (Supplementary Fig. 3b–d). In the liver tissues, there were no significant changes in mRNA expression of the genes related to inflammation (Tnf, Il1b, Il6, and Ccl2), fibrosis (Pdgfra, Timp1, Col1a1, and Tgfba) and lipid metabolism (Fasn, Srebp1, Ppara, Pparg, Cidec and Scarb1) in Gpnmb^−/−^ mice (Fig. 2f), it reflected the mild phenotype of Gpnmb^−/−^ mice fed with HFHS chow.

**Figure 3:**
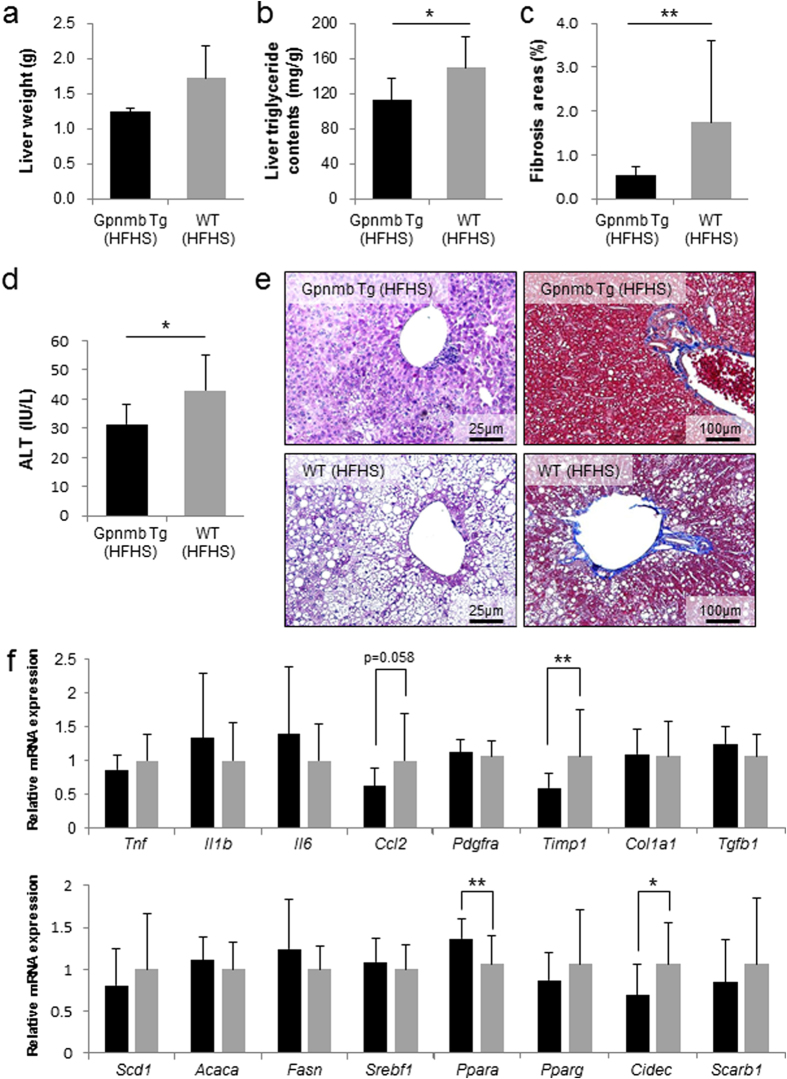
Amelioration of liver fibrosis and fat accumulation in Gpnmb Tg mice under HFHS chow. (**a,b**) Liver weight and triglyceride contents of Gpnmb Tg and WT mice under HFHS chow. (**c,d**) Liver fibrosis areas and serum ALT levels of Gpnmb Tg and WT mice under HFHS chow. (**e**) Periodic acid-Schiff (PAS) staining (left panels) and Azan-Mallory staining (right paels) of liver tissues of Gpnmb Tg and WT mice under HFHS chow. (**f**) Analysis of inflammation, fibrosis and fatty acid synthesis-related genes. mRNA levels of liver tissues of Gpnmb Tg and WT mice under HFHS chow by quantitative real-time PCR. All data are presented as mean ± standard deviation (SD) n = 8. *p < 0.05, **p < 0.01 vs. WT mice.

The phenotype of Gpnmb^−/−^ mice fed with HFHS chow was rather limited and we next produced Gpnmb Tg C57BL/6JJcl mice under the control of aP2 promoter (Supplementary Fig. 1c,a). We generated 3 lines of Gpnmb Tg mice, L-1, L-6 and L-14, in which Gpnmb mRNA expressions in epididymal fat were 70-, 7-, and 20-folds higher than WT mice. Glycosylated form of Gpnmb was abundantly expressed in epididymal WATs, but again all three lines of Gpnmb Tg mice demonstrated no discernible differences in body weight, fat pad mass and glucose metabolism under HFHS chow compared with WT mice (Supplementary Fig. 4b–g). Throughout the manuscripts, we presented the data derived from high expression Tg line, *i.e.* L-1. Serum total cholesterol, triglyceride levels were not altered in Gpnmb Tg mice (Supplementary Fig. 5a). However, liver weight tended to be reduced in Gpnmb Tg mice under HFHS chow (Fig. 3a), and the accumulation of lipid droplets and liver triglyceride contents were significantly suppressed in Gpnmb Tg mice (112.4 ± 25.0 v.s. 149.3 ± 35.5 mg/g, p = 0.03) (Fig. 3b). In Azan-Mallory stain, liver fibrosis areas were also significantly reduced (0.53 ± 0.19 v.s. 1.75 ± 1.85%, p = 6.72 × 10^−4^) and serum ALT levels were significantly lower in Gpnmb Tg mice compared with WT mice (31.5 ± 6.6 v.s. 43.0 ± 12.1 U/l) (Fig. 3c–e). Sirius Red staining demonstrated that fibrosis areas significantly reduced in Gpnmb Tg mice and liver hydroxyproline contents were also suppressed (Supplementary Fig. 5d–f). In Gpnmb Tg mice, the mRNA levels of Timp1 and Cidec were significantly decreased, and Ppara were significantly increased compared with WT mice (Fig. 3f). In addition, mRNA expression of Sod1 and Cat, anti-oxidative stress genes, were significantly increased in Gpnmb Tg mice and oxidative stress in the liver demonstrated by malondialdehyde (MDA) was ameliorated although it did not reach statistically significant levels (Supplementary Fig. 6).

### Gpnmb localizes in macrophages and stellate cells in the liver, and interacts with calnexin

**Figure 4:**
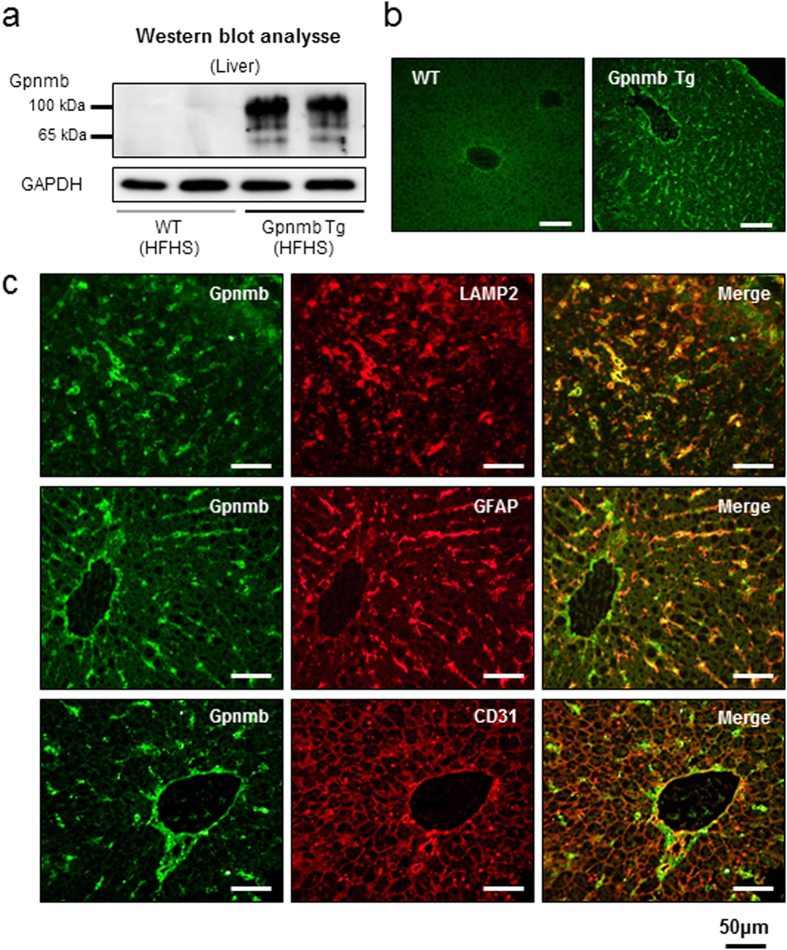
Expression of Gpnmb in macrophages and stellate cells in the liver. (**a**) Western blot analyses in liver of Gpnmb Tg and WT mice under HFHS chow. (**b**) Immunofluorescence staining of Gpnmb in Gpnmb Tg and WT mice. (**c**) Double immunofluorescence staining of LAMP2 and Gpnmb (upper panels), GFAP and Gpnmb (middle panels), CD31 and Gpnmb (lower panels).

mRNA expression of Gpnmb was predominantly enhanced in the epididymal adipose tissues in Gpnmb Tg mice (Supplementary Fig. 7a), however, the protective effects of Gpnmb was restricted to the liver and we next investigated the localization of Gpnmb in liver tissues. The robust band of 100 kDa glycosylated and minor 65 kDa unglycosylated forms were detected in Gpnmb Tg mice, while they were barely detectable in WT mice (Fig. 4a). Immunofluorescence staining also confirmed the presence of Gpnmb protein in the liver tissues of Gpnmb Tg mice and not in WT mice (Fig. 4b). Gpnmb protein mainly co-localized with lysosomal-associated membrane protein 2 (LAMP2), a macrophage marker, and glial fibrillary acidic protein (GFAP), a stellate cell marker. In addition, Gpnmb protein was partly co-localized with CD31, an endothelial cell marker, especially in the large vessels (Fig. 4c).

**Figure 5:**
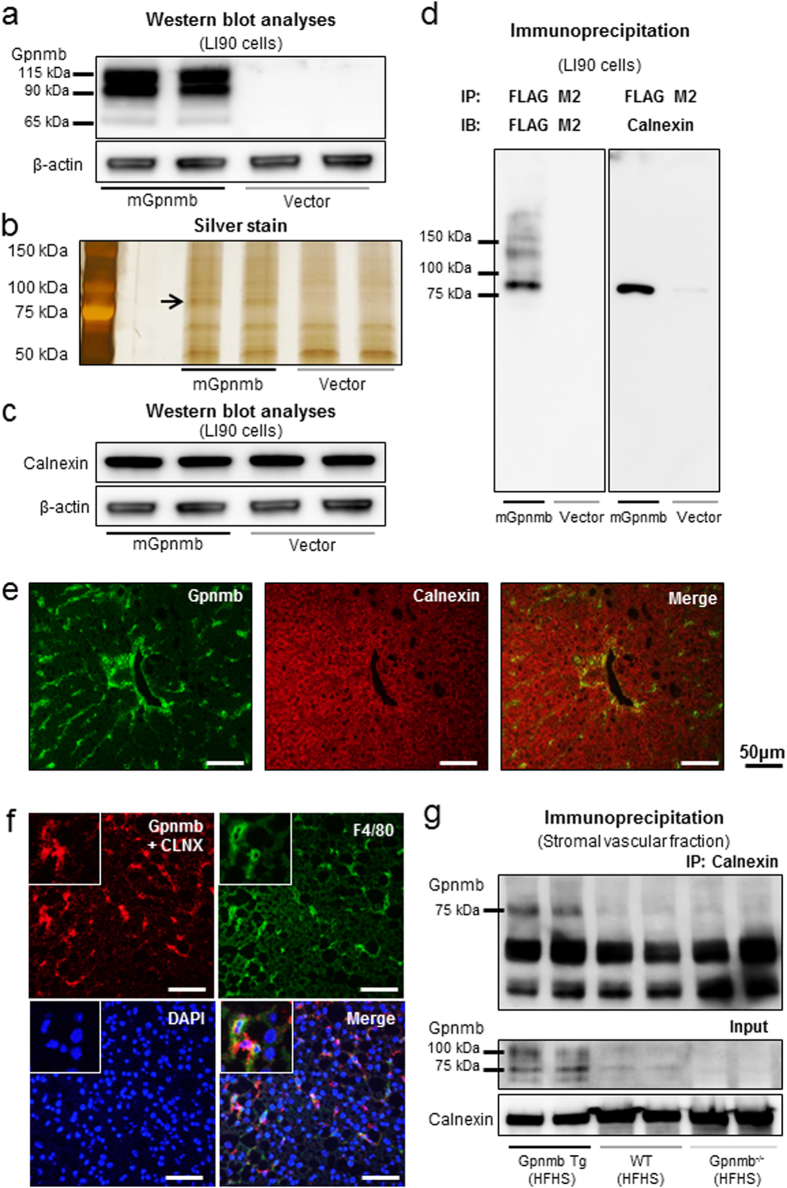
Interaction of Gpnmb with calnexin in hepatic macrophages and stellate cells of the liver. (**a**) Western blot analyses of LI90 cells transfected with p3xFLAG-mGpnmb or p3xFLAG-Vector by using anti-Gpnmb antibody. (**b**) Soluble proteins with FLAG tag were isolated and analyzed by SDS-PAGE and silver stain. Visible bands (black arrow) were excised and analyzed with LC-MS/MS, and calnexin was identified. (**c**) Western blot analyses re-probed with anti-calnexin antibody using the membranes in panel (**a**,**d**) Calnexin was co-immunoprecpitated with FLAG-tagged Gpnmb protein. (**e**) Double immunofluorescence staining of calnexin and Gpnmb. (**f**) We used Duolink proximity ligation assay to identify the interaction between Gpnmb and Calnexin. The red signals showing the interaction between Gpnmb and Calnexin were mainly detected in F4/80-positive cells. Scale bar: 50 μm. (**g**) Cell lysates derived from stromal vascular fractions were immunoprecipitated with anti-calnexin antibody and immunoblotted with anti-Gpnmb antibody.

To identify the interacting molecules of Gpnmb in macrophages and stellate cells, we performed the immunoprecipitation and subsequent LC-MS/MS analyses. We transfected p3xFLAG-mGpnmb plasmid to LI-90, a stellate cell line, and confirmed the protein expression of Gpnmb tagged with FLAG epitope by Western blotting analyses (Fig. 5a). Although both morphology of LI-90 cells and oxidative stress measured by dihydroethidium were not altered by the transfection of p3xFLAG-mGpnmb, mRNA expressions of COL1A1 and ACTA1 significantly reduced compared with control vector-treated LI-90 cells (Supplementary Fig. 8a–c). Then, soluble proteins were purified by anti-FLAG M2 affinity resin and the silver staining demonstrated the ~75 kDa bands which were not detected in a control vector treated LI-90 cells (Fig. 5b). The ~75 kDa bands were identified as calnexin by in-gel digestion and LC-MS/MS analysis. The expression of calnexin was demonstrated by Western blot analyses in both p3xFLAG-mGpnmb and control vector transfected LI-90 cells (Fig. 5c) and the complex of FLAG-tagged Gpnmb protein and calnexin was co-immunoprecpitated by anti-FLAG M2 affinity resin (Fig. 5d). We further confirmed the localization of the calnexin in the liver by immunohistochemistry. Calnexin diffusely expressed throughout the liver tissues and was partly co-localized with Gpnmb (Fig. 5e). We next used Duolink proximity ligation assay (PLA) to identify the protein-protein interaction between Gpnmb and calnexin. Liver tissue sections were incubated with goat anti-Gpnmb and rabbit anti-calnexin primary antibodies, followed by the application of a specially designed pair of oligonucleotide-conjugated secondary antibodies, PLA probes anti-goat PLUS and anti-rabbit MINUS. Red fluorescent signals are visualized by the formation of ligated closed circle and rolling-cycle amplification only when PLA PLUS and MINUS probes are in close proximity. The red signals were readily detected in the F4/80-positive macrophages (Fig. 5f). We also isolated the stromal vascular fractions containing the macrophages from epididymal adipose tissues. We confirmed the increase in the Gpnmb-calnexin complex formation in the Gpnmb Tg mice by immunoprecipitation (Fig. 5g). We also fractionated the plasma membrane and cytosolic fraction of liver tissues and Gpnmb mainly increased in the plasma membrane fractions of Gpnmb Tg mice (Supplementary Fig. 7b).

### Serum Gpnmb is a novel marker for nonalcoholic steatohepatitis (NASH)

**Figure 6:**
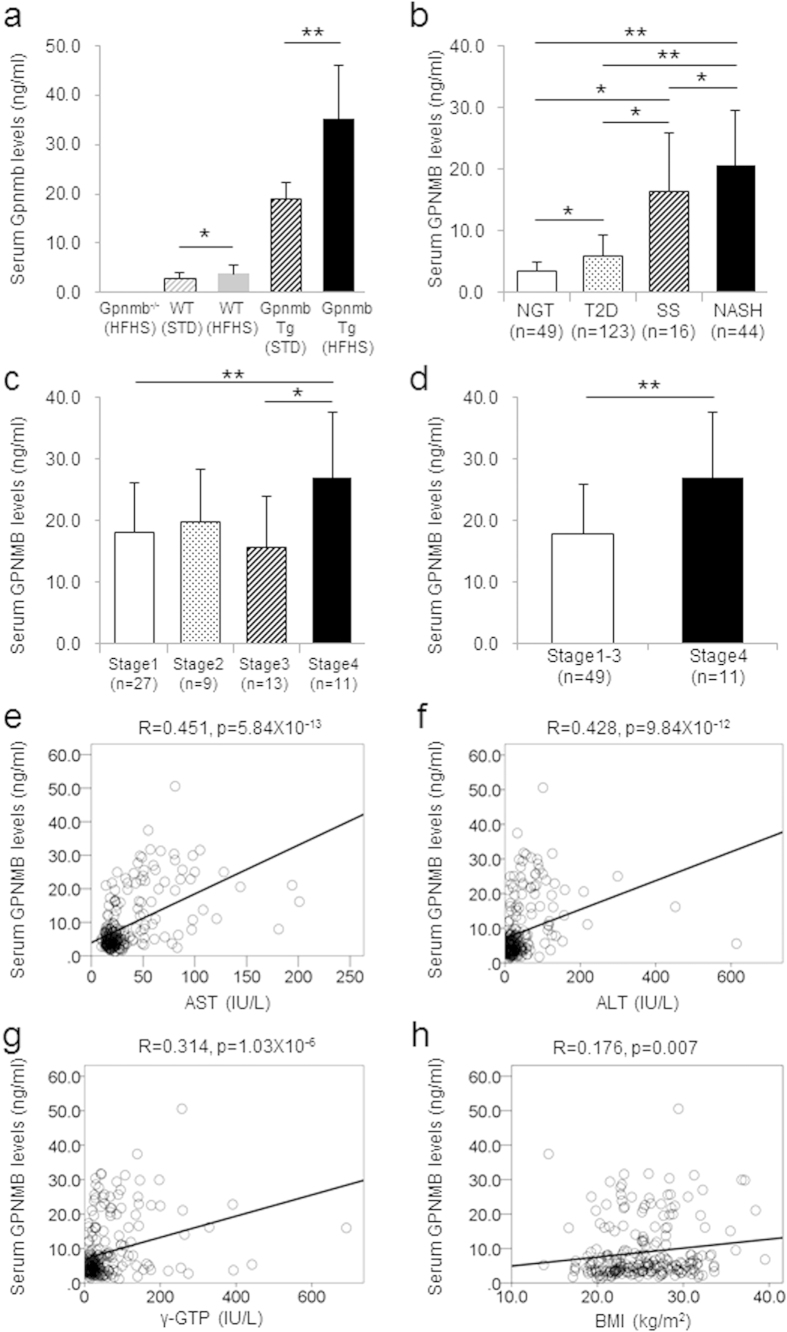
Elevation of serum GPNMB levels in the patients with fatty liver disease. (**a**) Serum Gpnmb levels of Gpnmb^−/−^, WT and Gpnmb Tg mice under HFHS and STD chow. n = 10–15. (**b**) Serum GPNMB levels in the subjects with normal glucose tolerance (NGT), the patients with type 2 diabetes (T2D), simple steatosis (SS) and nonalcoholic steatohepatitis (NASH). (**c**) Serum GPNMB levels in the patients with nonalcoholic fatty liver disease (NAFLD). They were classified into four groups (stage 1–4) according to the degree of liver fibrosis. (**d**) Serum GPNMB levels in the patients with NAFLD stage 4 (liver cirrhosis) and stage 1 to 3 (pre-cirrhosis). (**e–h**) The simple correlation of serum GPNMB with AST (**e**), ALT (**f**), γ-GTP (**g**), BMI (**h**) are shown. All data are presented as mean ± standard deviation (SD). *p < 0.05, **p < 0.01.

The findings in animal experiment indicate that the secreted and membrane-bound forms of Gpnmb were produced from both WATs, liver macrophages, and stellate cells along with the development of obesity, they mainly localize with the macrophages and stellate cells in the liver by binding to cell-surface calnexin, and the Gpnmb exerted the protective effects against the development of fatty liver disease. Indeed, soluble Gpnmb in the sera in Gpnmb^−/−^ mice was barely detected and serum Gpnmb levels were higher in mice under HFHS compared with STD chow in both WT mice (3.85 ± 0.35 v.s. 2.91 ± 0.30 ng/ml, p = 0.029) and in Gpnmb Tg mice (35.22 ± 2.46 v.s. 18.85 ± 1.15 ng/ml, p = 2.12 × 10^−9^) (Fig. 6a). In next attempt, we measured serum GPNMB levels in human. In the patients with type 2 diabetes (T2D), serum GPNMB levels were significantly higher than the subjects with normal glucose tolerance (NGT) (5.83 ± 3.49 v.s. 3.48 ± 1.48 ng/ml, p = 0.046). We also investigated the patients with liver biopsy-proven NAFLD, i.e. hepatic simple steatosis (SS) (n = 16, 48.8 ± 3.6 years) and nonalcoholic steatohepatitis (NASH) (n = 44, 53.3 ± 2.2 years) (Table 1). Serum GPNMB levels were prominently elevated in SS compared with NGT and T2D, and they were further elevated in NASH compared with SS (20.61 ± 9.00 v.s. 16.34 ± 9.53 ng/ml, p = 0.032) (Fig. 6b). We further classified the NAFLD patients into four groups according to the degree of liver fibrosis. Serum GPNMB levels showed the tendency to increase with the progression of liver fibrosis (stage 1; n = 27, 18.14 ± 7.99 ng/ml, stage 2; n = 9, 19.90 ± 8.42 ng/ml, stage 3; n = 13, 15.64 ± 8.29 ng/ml, stage 4; n = 11, 26.92 ± 10.76 ng/ml) (Fig. 6c). Serum GPNMB levels significantly increased in the patients with stage 4 manifesting liver cirrhosis compared with stages 1–3 without liver cirrhosis (26.92 ± 10.76 v.s. 17.80 ± 8.11 ng/ml, p = 0.005) (Fig. 6d). Since serum GPNMB levels were higher, and platelet counts and ALT levels were lower in stage 4, we performed multivariate logistic regression analysis to assess the parameters as a risk for liver fibrosis. As a result, serum GPNMB was an independent risk for stage 4 (Odds ratio, 1.109 [1.001–1.229], p = 0.049) (Table 2). Although serum GPNMB well-correlated with degree of liver fibrosis, it did not demonstrate significant correlations with NAFLD activity score (NAS) and necroinflammatory grading system, which represent the degrees of steatosis, ballooning and inflammation (Supplementary Fig. 9a,b). We also found the simple correlation of serum GPNMB levels with clinical parameters; serum AST (R = 0.451, p = 5.84 × 10^−13^), ALT (R = 0.428, p = 9.84 × 10^−12^), γ-GTP levels (R = 0.314, p = 1.03 × 10^−6^), and BMI (R = 0.176, P = 0.007), respectively (Fig. 6e–h, Table 2). The data suggested that serum *GPNMB* is a novel marker for the development and progression of NAFLD.

## Discussion

Although Gpnmb was initially identified from the osteoblasts, melanoma and various cancer cell lines, macrophages, and dendritic cells^11^, it is also expressed in WATs and liver. In the liver, CD68-positive sinusoid-lining cells were positive for Gpnmb mRNA by *in situ* hybridization, the isolated Kupffer cells were also positive for mRNA by Northern blot analysis, and the transcriptional activities increased during the *in vitro* culture^32^. In the acutely injured rat liver model by carbon tetrachloride (CCl_4_), Gpnmb mRNA was strongly increased in the pericentral inflammatory cells and sinusoid-lining CD68 positive cell^32^. In addition, Gpnmb mRNA was enhanced during the development of cirrhosis and hepatocellular carcinoma (HCC)^33^. Although the expression of Gpnmb is regulated by BMP-2 in osteoblasts, RANKL (receptor-activated NFκB ligand) in osteoclasts, CSF (colony stimulating factor) in hepatocellular carcinoma, endothelin-1 in melanoma cells and mTORC1 in adipocytes, the expression regulation of Gpnmb in hepatic macrophages and stellate cells under obesity is not elucidated in current investigation. The Gpnmb transgenic rats under the control of liver specific human serum amyloid P (SAP) promoter were protected from liver fibrosis under choline-deficient, L-amino acid-defined (CDAA) diet^34^. In contrast to liver, the expression of Gpnmb in WATs and its upregulation in obesity was recently found by Gabriel TL *et al.*^31^ and by us in the previous report as a clone name of OL-7^9^. The simple correlation between body weight and the expression of Gpnmb in both rodents and human was reported and the expression of Gpnmb in cultured RAW264.7 cells was induced by various lysosomal stress inducers such as palmitate and chloroquin or Torin1, an inhibitor of mammalian target of rapamycin complex 1 (mTORC1)^31^. Western blot analysis demonstrated that Gpnmb is abundantly expressed in 3T3-L1 preadipocytes, which suggested that both the adipose tissue macrophages and preadipocytes are major source of Gpnmb and the expression of Gpnmb is predominantly accentuated in adipose tissues compared with liver in mice under HFHS chow. Since aP2 promotor drives the transcriptional activities in both adipocytes and macrophages, we generated the aP2-driven Gpnmb Tg mice. However, we finally elucidated Gpnmb in macrophages plays critical role in steatohepatitis and ideally we should generate adipocyte-specific and macrophage-specific transgenic and knockout mice, which is one of the limitations of current investigation.

In current investigation, the body weight, fat pad weight, adipocyte size, glucose tolerance, insulin sensitivity, and tissue inflammation such as formation of crown-like structures (CLSs) in the WATs were not altered in Gpnmb^−/−^ mice. Interestingly, the overexpression of Gpnmb mainly ameliorated the fat accumulation and fibrosis in the liver. Since the inflammatory response and formation of CLSs in the WATs were not changed in Gpnmb Tg and Gpnmb^−/−^ mice under HFHS chow, we speculated that anti-oxidative action of Gpnmb is mainly involved in the amelioration of the fatty liver disease. In the mouse model of amyotrophic lateral sclerosis (ALS) carrying the mutant superoxide dismutase (SOD1^G93A^) and ALS patients, GPNMB was greatly induced during the disease progression and it was especially expressed in the motor neurons and astrocytes. In an NSC34 cell line, glycosylation of GPNMB was inhibited by the SOD1^G93A^, and the secretion of extracellular soluble form from activated astrocytes attenuated the neurotoxicity of SOD1^G93A^ in neural cells^35^. Authors speculated that both reduced production of mature and glycosylated forms of GPNMB in motor neuron and suppressed shedding of soluble GPNMB may rescue the neurons by inhibiting apoptosis. In addition, GPNMB is also neuroprotective in ischemia-reperfusion model in rats by enhancing the phosphorylation of extracellular signal-regulated kinase 1 and 2 (ERK1/2) and protein kinase B (Akt)^36^. We found the increase in the mRNA expression of Sod1 and Cat and reduction of malondialdehyde (MDA) concentration in the liver tissues of Gpnmb Tg under HFHS chow, suggesting Gpnmb reduces the oxidative stress in the liver.

To elucidate the molecular mechanism for the anti-oxidative properties of Gpnmb in macrophages and stellate cells, we identified calnexin as an interacting protein with Gpnmb. Calnexin is a molecular chaperone, type 1 membrane-bound protein in endoplasmic reticulum (ER), and participates in the folding and assembly of the nascent proteins. Calnexin interacts with newly synthesized glycoproteins through monoglucosylated oligosaccharides to regulate the quality control of glycoproteins in the ER. Thus, we hypothesized that the transgenic expression of Gpnmb in Kupffer and stellate cells in liver exerts anti-oxidative actions by binding calnexin in ER and ameliorates the liver fibrosis. Recently, it has been reported that ER stress induced fibrogenic activity in hepatic stellated cells through apoptosis and autophagy mechanism^37^,^38^,^39^,^40^. In current study, we demonstrated the prominent expression of Gpnmb in hepatic macrophages and stellate cells, and proximity ligation assay (PLA) clearly demonstrated that direct interaction between Gpnmb and calnexin in the hepatic macrophages. Although Gpnmb did not alter the ER stress markers (data not shown), it increased anti-oxidative markers and reduced the oxidative stress in the liver tissues in current investigation.

In this study, we also emphasize the importance of circulating GPNMB in fatty liver disease as a biomarker and therapeutic target (Supplementary Fig. 12). In Gpnmb Tg mice under HFHS chow, Gpnmb mRNA expression in WATs was 55-fold higher compared with liver and WATs are major source of circulating Gpnmb. We further investigated the concentration of soluble Gpnmb in the sera derived from the experimental mice. Circulating Gpnmb was increased in Gpnmb Tg mice and further accentuated by HFHS chow. Vast amount of soluble Gpnmb produced in WATs flowed into the liver and it exerted the anti-oxidative action against the fatty liver disease. Under ER stress, calnexin was also reported to locate on cell surface in various cell types, such as thymocytes and neurons^41^,^42^,^43^. We speculated that the soluble forms of Gpnmb derived from WATs bind cell-surface calnexin expressed in Kupffer and stellate cells. Actually, both Gpnmb and calnexin were demonstrated in the plasma membrane fractions and plasma membrane-bound Gpnmb was prominently enhanced in the liver of Gpnmb Tg mice. We further extended the research to clinical applications of the measurement of serum GPNMB in the patients with NAFLD. In NAFLD, the liver biopsy is gold standard for the diagnosis of NASH, but useful biomarkers for NASH are required in the management of NAFLD. We demonstrated that serum soluble GPNMB levels are statistically higher in the patients with NASH compared with the patients with SS and it may be useful for the diagnosis for the presence of NASH. Since the levels of serum soluble Gpnmb were far higher than control subjects and the patients with ALS previously described^35^, the elevation of serum GPNMB is specific for the development of NASH. In addition, serum GPNMB levels correlated with the severity of fibrosis in the liver and also with liver function tests such as AST, ALT, and γGTP.

Although soluble Gpnmb may be beneficial in the diagnosis and possible therapeutic target of fatty liver disease, brain ischemic injury and ALS, one of the concerns is the possible progression of various malignancies in the clinical application. The high expression of GPNMB is related to the recurrence of breast cancer^44^, melanoma^25^ and pancreatic cancers^45^. The enhanced migration properties were observed in various malignant cell lines such as in glioma and hepatoma cell lines. Gpnmb enhanced MMP-3 expression in 4TI-mouse mammary carcinoma cells, suggesting the role of Gpnmb in tumor aggressiveness^44^. However, there also contradictory reports suggesting Gpnmb may act as tumor suppressor in breast cancer cell lines. For example, tumor suppressor protein p53 interacts with Gpnmb pomotor and up-regulates its expression^32^,^46^. Thus, more studies are required to confirm the role of Gpnmb in the progression of various malignancies.

There are some limitations in current investigation. Since aP2 promotor drives the transcriptional activities in both adipocytes and macrophages, we generated the aP2-driven Gpnmb Tg mice. However, we finally elucidated Gpnmb in macrophages plays critical role in steatohepatitis and ideally we should generate adipocyte-specific and macrophage-specific transgenic and knockout mice, which is one of the limitations of current investigation. Since Gpnmb^−/−^ mice were generated by backcrossing DBA2J mice with naturally occurring stop codon mutation of Gpnmb to C57BJ mice, mutated short form of Gpnmb may not be detected anti-Gpnmb antibody, which recognizes extracellular domain expanding from Lys23-Asn502. Undetected short form of Gpnmb may influenced the phenotypes of Gpnmb^−/−^ mice. DBA2J mice carry the mutations of several genes besides Gpnmb. Therefore, we can’t completely negate the possibility in which phenotypes observed in Gpnmb^−/−^ mice are influenced by the mutations of other genes, although we backcrossed DBA2J mice to C57BJ mice more than 10 generations.

In conclusion, the overexpression of Gpnmb in hepatic macrophages and stellate cells ameliorated the oxidative stress in liver and demonstrated therapeutic impacts on the development of fatty liver disease and fibrosis in mouse model of diet-induced obesity. In addition to *in situ* overexpression of Gpnmb in the liver, the direct influx of soluble Gpnmb derived from visceral adipose tissues also contributed to the amelioration of fatty liver disease. In the patients with NASH, soluble GPNMB concentrations in sera were higher compared with the patients with SS and soluble GPNMB is a promising biomarker and therapeutic target for the development and progression of NAFLD in obesity.

### Experimental procedures

#### Generation of *Gpnmb* transgenic (Tg) and deficient mice

Gpnmb Tg mice (Accession No. CDB0503T: http://www.clst.riken.jp/arg/TG%20mutant%20mice%20list.html) in the background of C57BL/6JJcl were generated as described below. aP2 promoter and β-globin intron were amplified by PCR primers, 5′-GGGGGGCTCGAG(*Xho*I)CCAGCAGGAATCAGGTAGCTGGAGA-3′ and 5′-GGGGGGGTCGAC(*Sal*I)GAATTC(*EcoR*I)TTTGCCAAAATGATGAGACAGCACA-3′ and ligated into *Xho*I and *Sal*I site of pBluescript KS( + ) (Stratagene, Santa Clara, CA). Next, human GH poly A signal was amplified by primers, 5′-GGTCGAC(*Sal*I)ATCGAT(*Cla*I)GCATGC(*Sph*I)GAATTC(*EcoR*I)ACTCCTCAGGTGCAGGCTGC-3′ and 5′-GGGGGGGCGGCCGC(*Not*I)GGATCTCGATCTTCATAAGAGAAGA-3′ and ligated *Sal*I and *Not*I site of the previous plasmid. Finally, full-coding cDNA of Gpnmb was amplified by primers, 5′-GGGGGGGTCGAC(*Sal*I)TCGGAGTCAGCATGGAAAGTC-3′ and 5′-GGGGGGGCATGC(*Sph*I)GTCAGAGGGAAGGCCAAAGAC-3′, and ligated into *Sal*I and *Sph*I site of the construct (Fig. S1c). The insert was excised with *Xho*I and *Not*I and the transgene was generated. Microinjected C57BL/6NJcl one-cell stage zygotes were oviduct-transferred and permitted to develop to term and the three transgenic founders were obtained by genotyping. For genotyping, the tails of 0.3–0.4 cm length were cut from 5-week-old mice and genomic DNAs were extracted by using DNeasy Blood & Tissue Kit (QIAGEN, Hilden, Germany) after the digestion with Proteinase K in Buffer ATL for 12 hours. Transgenes were amplified by PCR by using PrimeSTAR GXL DNA Polymerase (TaKaRa) and primer sets, 5′-CAGTCAACTTGACAGCTGGC-3′ (Gpnmb-sequence-S1) and 5′-GCATGATGCTGACTTCCAGG-3′ (GpnmbAS1); 5′-CACTCCTACAGTCACATGGTCAG-3′ (AP2) and 5′-CGAAATCGCTTGGCAGCCTG-3′ (Gpnmb-Tg-AS) (Fig. S1d).

Since DBA2J mice have the stop codon mutation with Gpnmb (R150X)^20^, we generated Gpnmb deficient mice by using DBA/2J mice. Male DBA2J mice were mated with C57BL/6JJcl mice to generate heterozygous Gpnmb^+/−^ mice, and they were backcrossed to C57BL/6JJcl mice for more than ten generations. Genotyping of Gpnmb^−/−^ mice was performed by PCR using primers 5′-CTTGGCTGTGATACCAGTGAGTGAG-3′ and 5′-CTCATTGTATGCCTGTGGTGTCCTG-3′, and by cutting with *Pvu*II after PCR.

### Animal experiments

For expression studies, 4-week-old Otsuka Long-Evans Tokushima fatty (OLETF) and Long-Evans Tokushima Otsuka (LETO) rats were housed in a room with 12-h light/dark cycles, and the animals were allowed free access to food and water. OLETF rats were divided into four groups, with 15 animals in each group: (i) control OLETF rats; (ii) OLETF rats receiving pioglitazone (TZD, 1 mg/kg body weight per day); (iii) OLETF rats subjected to voluntary exercise (EXE, access to running wheel); and (iv) insulin-treated OLETF rats (INS). In the INS group, neutral protamine Hagedorn insulin (10–40 units/animal) was administered to maintain fasting glucose levels of 80–150 mg/dl. Five animals in each group were killed after overnight fasting, and various tissues were harvested at 6, 30, and 50 weeks of age. White adipose tissues (WATs) were digested with collagenase in Krebs-Ringer Hepes buffer, and mature adipocytes and vascular stromal cells were separated and subjected to RNA isolation and immunoblotting^47^.

Male mice were maintained under a 12-hour light-dark cycle with free access to chow and drinking water. From 5 weeks of age, the Gpnmb^−/−^, Gpnmb^+/+^, Gpnmb Tg and wild type (WT) mice were fed with high-fat, high-sucrose (HFHS) (D12331, Research Diet) or standard (STD) chow (NMF, Oriental Yeast) (n = 8–10 each group). For glucose tolerance test (GTT), the mice at 15 weeks of age were fasted for 16 hours with free access to drinking water. After determination of fasting blood glucose levels, the mice were intraperitoneally injected with 1.0 g glucose per kg body weight. Insulin tolerance test (ITT) was carried out on fed mice at 15 weeks of age by intraperitoneal injection of human insulin 1.0 U per kg body weight for HFHS groups. Mice were finally sacrificed for the following experiments at 25 weeks of age. Liver triglyceride was measured by Folch method (Skylight Biotech, Tokyo, Japan). Liver hydroxyproline was measured by QuickZyme hydroxyproline assay (QuickZyme Biosciences, Leiden, Netherlands). Serum Gpnmb levels were measured with ELISA kit for mouse Osteoactivin/Gpnmb (R&D Systems, Minneapolis, MN). The methods were carried out in accordance with the approved guidelines. All experimental protocols were approved by the Animal Care and Use Committee of the Department of Animal Resources, Advanced Science Research Center, Okayama University.

### Cell culture

LI90 (human liver stellate cell) cells were obtained from Japanese collection of research bioresources (JCRB). LI90 cells were cultured in Dulbecco's modified Eagle's medium (DMEM) (Life technologies) with 10% fetal bovine serum and 100 units/mL streptomycin. Mouse 3T3-L1 fibroblasts (DS pharma biomedical) were cultured *in vitro* and differentiated into mature adipocytes. 3T3-L1 cells were grown in 3T3-L1 Preadipocyte Medium (ZenBio) and propagated to confluence. Forty-eight hours later, designated as Day 0, the adipocyte differentiation was initiated using 3T3-L1 Differentiation Medium (ZenBio). Three days after induction, the medium was replaced with 3T3-L1 Adipocyte Maintenance Medium (ZenBio).

### Northern blot analysis

Two micrograms of mRNA extracted from visceral and subcutaneous adipose tissue of LETO and OLETF rats was subjected to 2.2 M formaldehyde-1% agarose gel electrophoresis and capillary transferred to the Hybond N + nylon membranes (Amersham). Plasmid clones were digested with *EcoR*I, and the inserts were purified by QIAEX kit (Qiagen), and radiolabeled with [α-^32^P]dCTP, using the random primer labeling system (Amersham). The membranes were hybridized with radiolabeled cDNA probes (1 × 10^6^ cpm/ml) at 42°C in 50% formamide, 5 × SSC (saline sodium citrate), 1 × Denhardt's solution, 50 mM sodium phosphate (pH 7.0), and salmon sperm DNA at 200 μg/ml for 24 h. Filters were washed under high-stringency conditions (four times at room temperature for 15 min in 1 × SSC-0.1% SDS, followed by two times at 50°C in 0.1 × SSC-0.1% SDS). β-actin served as an internal control.

### Western blot analysis

Liver and adipose tissues were excised and homogenized with RIPA (Radio-Immunoprecipitation Assay) buffer (Thermo Fisher Scientific, Rockford, IL). Cell culture pellets of 3T3-L1 and LI90 cells were homogenized with CelLytic M with Protease Inhibitor Cocktail (Sigma-Aldrich, St. Louis, MO). After centrifugation at 14,000 rpm for 30 min at 4 °C, supernatants were collected for the further analyses. Equal amounts of protein were subjected to SDS-PAGE under reducing conditions, and were electroblotted onto Hybond P polyvinylidine fluoride membranes (GE Healthcare Life Sciences, Pittsburg, PA). The membrane blots were immersed in a blocking solution containing 5% nonfat dry milk and Tris-buffered saline with Tween-20 (0.05% Tween-20, 20 mM Tris-HCl, and 150 mM NaCl, pH 7.6). Then, the membranes were incubated with the following primary antibodies: goat polyclonal anti-Osteoactivin/Gpnmb (R&D Systems), rabbit polyclonal anti-Calnexin (Abcam; ab22595), rabbit monoclonal anti-GAPDH (D16H11), and anti-β-actin (D6A8) (Cell Signaling Technology, Beverly, MA). The blots were washed three times with Tris-buffered saline with Tween-20, immersed in Pierce ECL Plus Substrate (Thermo Fisher Scientific), and then the chemiluminescence was analyzed using the LAS-3000 mini instrument (FUJIFILM, Tokyo, Japan).

### Morphological studies

Liver and epididymal adipose tissue specimens were fixed in 10% formaldehyde and embedded in paraffin, and 4 μm-thick sections were prepared. The sections were stained with periodic acid-Schiff (PAS), and only liver sections were stained with Azan-Mallory and Sirius red. To evaluate the adipocyte size and hepatic fibrosis areas, we examined 10 randomly selected fields per animal at 20 weeks after the loading of HFHS diet. The areas of hepatic fibrosis and adipocyte size were measured by using the Lumina Vision software program (Mitani Corporation, Tokyo, Japan). Frozen liver sections were fixed in cold acetone for 3 min and incubated with goat polyclonal anti-Osteoactivin/Gpnmb antibody (R&D Systems) overnight at 4 °C. Next they were stained with Alexa Fluor 488-labeled donkey anti-goat IgG (Life Technologies) for 1 h at 22 °C. Frozen sections were double stained for LAMP2, GFAP, CD31 and Calnexin by indirect immunofluorescence. Serial sections (4 μm) were incubated with Anti-LAMP2 antibody (abcam; ab13524), Anti-CD31 antibody (abcam; ab28364), Anti-GFAP antibody (abcam; ab16997), Anti-Calnexin antibody (abcam; ab22595) and goat polyclonal anti-Osteoactivin/Gpnmb antibody (R&D Systems) at 22 °C for 1 h. Sections were then stained with Alexa Fluor 488-labeled donkey anti-goat IgG and Alexa Fluor 555-labeled goat anti-rabbit IgG or Alexa Fluor 555-labeled goat anti-rat IgG (Life Technologies) for 1 h, respectively.

### Real-time RT-PCR

Briefly, liver tissues were homogenized by Tissuelyser Adapter Set 2 × 24 (QIAGEN) with 1 ml QIAzol Lysis Reagent (QIAGEN) and total RNAs extracted by RNeasy Lipid Tissue Mini Kit (QIAGEN). Then, 1 μg total RNAs were used to synthesize cDNA by using High Capacity RNA-to-cDNA Kit (Applied Biosystems). Quantitative real-time PCR was performed in Step One Plus Real-Time PCR System (Applied Biosystems) with specific primers and Universal Master Mix II (Life Technologies). The relative abundance of mRNAs was standardized with 36B4 mRNA as the invariant control.

### Immunoprecipitation assay and LC-MS/MS (high performance liquid chromatography-tandem mass spectrometry)

Recombinant mouse Gpnmb lacking signal peptide tagged with 3xFLAG peptides (p3xFLAG-mGpnmb) were prepared by FLAG^®^ Protein Expression system (Sigma-Aldrich). p3xFLAG-mGpnmb and control vector were transfected to LI90 cells by Lipofectamine^®^ LTX Reagent (Life Technologies). The cells were stained with anti-FLAG M2 antibody and dihydroethidium (Sigma-Aldrich). Soluble proteins were purified by ANTI-FLAG M2 affinity resin (ANTI-FLAG M2 Affinity Gel, Sigma-Aldrich) and subjected to SDS-PAGE and silver staining. Visible bands were excised and in-gel-digested with trypsin and analyzed with LC-MS/MS (APRO life Science Institute, Inc, Tokushima, Japan).

### Duolink proximity ligation assay

Duolink proximity ligation assay (PLA) (Sigma-Aldrich) was used to identify the interaction between Gpnmb and Calnexin in the frozen liver sections of Gpnmb Tg mice. Frozen liver sections were fixed, blocked and incubated with goat anti-Gpnmb and rabbit anti-calnexin primary antibodies. We next applied the PLA probe anti-goat PLUS (DUO92003) and PLA probe anti-rabbit MINUS (DUO92005). Proximity ligation and polymerase amplification were then conducted *in situ* according to the manufacturer’s instructions. After polymerase amplification step, we incubated with Anti-F4/80 antibody (ab6640) for 1 h at 22 °C. The sections were then stained with Alexa Fluor 488-labeled donkey anti-rat IgG for 1 hour.

### Patients

Japanese subjects with normal glucose tolerance (NGT) (n = 49, 45.7 ± 9.1 years) and the patients with type 2 diabetes (T2D) (n = 123, 60.8 ± 14.4 years) were enrolled into this study. Furthermore, the patients with nonalcoholic fatty liver disease (NAFLD) who had been performed liver biopsy (n = 60, 52.1 ± 14.8 years) were also enrolled into this study. NAFLD patients were divided into two groups, hepatic simple steatosis (SS) (n = 16, 48.8 ± 3.6 years) and nonalcoholic steatohepatitis (NASH) (n = 44, 53.3 ± 2.2 years) by Matteoni’s classification of NAFLD^48^. A histological “staging” system was used to categorize the NAFLD patients in stages 1 to 4 according to the severity of liver fibrosis^49^. NASH activity score^50^ and necroinflammatory grading system^51^ were used for the evaluation of steatosis, hepatocellular ballooning and lobular inflammation. We obtained the written informed consent from each patient. The study was conducted in accordance with the ethical principle of the Declaration of Helsinki and approved by ethical committee of Okayama University Graduate School of Medicine, Dentistry and Pharmaceutical Sciences (#736).

### Blood sampling and assays

We measured overnight fasting serum levels of total cholesterol and low density lipoprotein (LDL) cholesterol, high density lipoprotein (HDL) cholesterol, triglycerides (L Type Wako Triglyceride·H, Wako Chemical, Osaka), aspartate aminotransferase (AST), alanine aminotransferase (ALT), γ-Glutamyltranspeptidase (γ-GTP) and HbA1c. Serum GPNMB levels were measured with ELISA kit for human Osteoactivin/Gpnmb (R&D Systems).

### Statistical analysis

All data are expressed as the means ± standard deviation (SD). For comparison of two groups, Mann-Whitney U test was used. The multiple comparisons were performed by a Kruskal-Wallis. Spearman correlation coefficients were used to evaluate whether serum Gpnmb levels correlated with various clinical parameters. A value of P < 0.05 was regarded as statistically significant. The data were analyzed using the IBM SPSS Statistics software program 22 (IBM, Armonk, NY).

## Additional Information

**How to cite this article**: Katayama, A. *et al.* Beneficial impact of Gpnmb and its significance as a biomarker in nonalcoholic steatohepatitis. *Sci. Rep.* **5**, 16920; doi: 10.1038/srep16920 (2015).

## Supplementary Material

Supplementary Information

## Figures and Tables

**Table 1:** Clinical characteristics in the patients with type 2 diabetes (T2D) and nonalcoholic fatty liver disease (NAFLD), and subjects with normal glucose tolerance (NGT).

			**NAFLD**		Total
	NGT	T2D	SS	NASH	
Number (male/female)	49 (27/22)	123 (73/50)	16 (8/8)	44 (17/27)	232 (124/108)
Age (years)	45.7 ± 9.1	60.8 ± 14.4**	48.8 ± 14.4^††^	53.3 ± 14.9*^,†^	55.4 ± 14.8
Height (cm)	164.0 ± 10.3	162.6 ± 9.5	161.3 ± 9.4	158.9 ± 10.2	162.1 ± 9.9
BW (kg)	65.0 ± 15.3	66.2 ± 14.5	68.7 ± 13.3	68.8 ± 15.7	66.6 ± 14.8
BMI (kg/m^2^)	24.0 ± 4.0	24.9 ± 4.3	26.5 ± 5.1	27.1 ± 4.9**^,†^	25.2 ± 4.5
HbA1c (%)	6.04 ± 0.39	7.35 ± 0.99**	5.40 ± 0.42^††^	5.80 ± 1.13^††^	6.64 ± 1.18
AST (IU/L)	21.9 ± 8.7	22.9 ± 12.8	44.0 ± 19.3**^,††^	74.8 ± 43.1**^,††,§§^	34.0 ± 29.9
ALT (IU/L)	23.9 ± 17.0	28.8 ± 56.1	67.1 ± 38.6*^,†^	95.9 ± 79.3**^,††^	43.1 ± 61.2
γ-GTP (IU/L)	31.8 ± 23.5	44.1 ± 64.3	98.9 ± 75.8**^,†^	115.1 ± 121.1**^,††^	58.8 ± 79.9
T-Cho (mg/dl)	211.1 ± 27.2	189.3 ± 40.2**	201.4 ± 46.7	192.0 ± 45.3	195.5 ± 40.1
TG (mg/dl)	91.4 ± 54.1	133.2 ± 68.5**	200.1 ± 151.0**^,††^	156.7 ± 81.5**	133.5 ± 81.3
HDL-C (mg/dl)	77.4 ± 21.9	61.9 ± 20.0**	56.3 ± 12.7**	54.6 ± 18.3**	63.5 ± 21.1
LDL-C (mg/dl)	125.2 ± 27.7	106.8 ± 29.1*	114.2 ± 46.6	119.3 ± 44.9	112.8 ± 33.4
GPNMB (ng/ml)	3.48 ± 1.48	5.83 ± 3.49*	16.34 ± 9.52**^,††^	20.61 ± 9.00**^,††,§^	8.86 ± 8.31

SS, hepatic simple steatosis; NASH, nonalcoholic steatohepatitis; BW, body weight; BMI, body mass index; AST, aspartate aminotransferase; ALT, alanine aminotransferase; γ-GTP, γ-Glutamyltranspeptidase; T-Cho, Total cholesterol; TG, Triglyceride; HDL-C, HDL cholesterol; LDL-C, LDL cholesterol; *p < 0.05; **p < 0.01 vs. NGT; ^†^p < 0.05; ^††^p < 0.01 vs. T2D; ^§^p < 0.05^; §§^p < 0.01 vs. SS. All data are presented as mean ± standard deviation (SD).

**Table 2:** Multivariate logistic regression analysis to assess the parameters as a risk for liver fibrosis (stage 4).

Risk factor for Liver fibrosis	B	Standard error	p	Odds ratio (95% confident intervals)	Predictive accuracy
Platelet (×10^4^/μl) (1 ×10^4^/μl increments)	−0.175	0.072	0.015*	0.840 (0.730–0.966)	
ALT (IU/L) (1 IU/L increments)	−0.037	0.019	0.048*	0.963 (0.928–1.000)	90%
Gpnmb (ng/ml) (1 ng/ml increments)	0.103	.052	0.049*	1.109 (1.001–1.229)	

ALT, alanine aminotransferase; *p < 0.05.
