# Supplementary material for: Beneficial impact of Gpnmb and its significance as a biomarker in nonalcoholic steatohepatitis
Source: Sci Rep. 2015 Nov 19;5:16920. doi: 10.1038/srep16920 (PMC4652285; doi:10.1038/srep16920)

Beneficial impact of Gpnmb and its significance as a biomarker in nonalcoholic steatohepatitis

Akihiro Katayama[1], Atsuko Nakatsuka[1], Jun Eguchi[1], Kazutoshi Murakami[1, 2], Sanae Teshigawara[1], Motoko Kanzaki[1], Tomokazu Nunoue[1], Kazuyuki Hida[3], Nozomu Wada[4], Tetsuya Yasunaka[4], Fusao Ikeda[4], Akinobu Takaki[4], Kazuhide Yamamoto[4], Hiroshi Kiyonari[5, 6], Hirofumi Makino[1], and Jun Wada[1]

[1]Department of Medicine and Clinical Science, Okayama University Graduate School of Medicine, Dentistry and Pharmaceutical Sciences, Kita-ku, Okayama 700-8558, Japan

[2]Department of General Medicine, Okayama University Graduate School of Medicine, Dentistry and Pharmaceutical Sciences, Kita-ku, Okayama 700-8558, Japan

[3]Department of Diabetes and Metabolism, National Hospital Organization Okayama Medical center, Kita-ku, Okayama 701-1154, Japan

[4]Department of Gastroenterology and Hepatology, Okayama University Graduate School of Medicine, Dentistry and Pharmaceutical Sciences, Kita-ku, Okayama 700-8558, Japan

[5]Animal Resource Development Unit and [6]Genetic Engineering Team, RIKEN Center for Life Science Technologies, 2-2-3 Minatojima Minami, Chuou-ku, Kobe 650-0047, Japan

Supplementary Figure legends

Supplementary Figure 1 mRNA expression levels of *Gpnmb* in OLETF rats and generation of Gpnmb transgenic (Tg) mice. a. Northern blot analyses in various tissues in obese OLETF rats (O) and lean LETO rats (L). **b.** Western blot analyses of mature adipocytes and stromal vascular fraction (SVF) in wild type (WT), Gpnmb Tg and $Gpnmb^{-/-}$ mice under high fat high sucrose (HFHS) chow. **c.** Schematic diagram showing structure of transgene consisting of aP2 promotor, β-globin intron, coding region of Gpnmb cDNA and hGH polyA tail. Primers, AP2, Gpnmb-Tg-AS, Gpnmb-sequence-S1 and GnnmbAS1 were used for the screening for the Gpnmb Tg mice. **d.** Gpnmb Tg mice #1, #12 and #13 demonstrated 824 and 600 bp PCR products using primer sets indicated in panel **c**.

Supplementary Figure 2 Body weight (BW), fat pad weight and glucose metabolism in $Gpnmb^{-/-}$ mice. a. Western blot analyses in epididymal fat of $Gpnmb^{-/-}$ and $Gpnmb^{+/+}$ mice under high fat-high sucrose (HFHS) chow. **b.** Body weight of $Gpnmb^{-/-}$ and $Gpnmb^{+/+}$ male C57BL/6JJcl mice fed with HFHS and standard (STD) chow. **c.** Fat pad weight of $Gpnmb^{-/-}$ and $Gpnmb^{+/+}$ mice at 25 weeks of age. **d.** The size distribution and the average size of adipocytes in epididymal adipose tissues of $Gpnmb^{-/-}$ and $Gpnmb^{+/+}$ mice under HFHS chow. **e.** Periodic acid-Schiff (PAS) staining of epididymal adipose tissues of $Gpnmb^{-/-}$ and $Gpnmb^{+/+}$ mice under HFHS chow. **f and g.** Glucose and insulin tolerance test of $Gpnmb^{-/-}$ and $Gpnmb^{+/+}$ mice under HFHS chow at 15 weeks of age. All data are presented as mean ± standard deviation (SD). n=8.

Supplementary Figure 3 Serum lipid profile and evaluation of liver fibrosis in Gpnmb-/- and Gpnmb+/+ mice. a. Total cholesterol, triglyceride, and LDL cholesterol in sera of $Gpnmb^{-/-}$ and $Gpnmb^{+/+}$ mice fed HFHS chow. **b.** Sirius red staining of liver tissues of $Gpnmb^{-/-}$ and $Gpnmb^{+/+}$ mice fed HFHS chow. **c.** Liver fibrosis areas of $Gpnmb^{-/-}$ and $Gpnmb^{+/+}$ mice fed HFHS chow. **d.** Liver hydroxyproline contents of $Gpnmb^{-/-}$ and $Gpnmb^{+/+}$

mice fed HFHS chow. All data are presented as mean ± standard deviation (SD). n=5. **p<0.01 vs. Gpnmb$^{+/+}$ mice.

Supplementary Figure 4 Body weight (BW), fat pad weight and glucose metabolism in Gpnmb transgenic (Tg) mice. a. Western blot analyses in epididymal fat of Gpnmb Tg and wild type (WT) mice under HFHS chow. **b.** Body weight of Gpnmb Tg and WT male C57BL/6JJcl mice fed with HFHS and STD chow. **c.** Fat pad weight of Gpnmb Tg and WT mice at 25 weeks of age. **d.** The size distribution and the average size of adipocytes in epididymal adipose tissues of Gpnmb Tg and WT mice under HFHS chow. **e.** Periodic acid-Schiff (PAS) staining of epididymal adipose tissues of Gpnmb Tg and WT mice under HFHS chow. **f and g.** Glucose and insulin tolerance test of Gpnmb Tg and WT mice under HFHS chow at 15 weeks of age. All data are presented as mean ± standard deviation (SD). n=8.

Supplementary Figure 5 Serum lipid profile and evaluation of liver fibrosis in Gpnmb Tg and WT mice. a. Total cholesterol, triglyceride, and LDL cholesterol of Gpnmb Tg and WT mice fed HFHS chow. **b.** Sirius red staining of liver tissues of Gpnmb Tg and WT mice fed HFHS chow. **c.** Liver fibrosis areas of Gpnmb Tg and WT mice fed HFHS chow. **d.** Liver hydroxyproline contents of Gpnmb Tg and WT mice fedHFHS chow. All data are presented as mean ± standard deviation (SD). n=8. **p<0.01 vs. WT mice.

Supplementary Figure 6 Quantitative RT-PCR in liver tissues. a. mRNA expression of *Sod1, Sod2, Cat, Gpx1, Cybb, and Ncf1* were not altered in Gpnmb$^{-/-}$ and Gpnmb$^{+/+}$ mice fed with high fat high sucrose (HFHS) chow. **b.** mRNA expression of *Sod1* and *Cat*, anti-oxidative stress genes, were significantly increased in Gpnmb Tg mice. **c and d.** The oxidative stress in the liver demonstrated by malondialdehyde (MDA) was ameliorated in Gpnmb Tg mice compared with wild type (WT) mice fed with HFHS chow. All data are

presented as mean ± standard deviation (SD). n=8. *p<0.05, **p<0.01 vs. WT mice.

Supplementary Figure 7 mRNA and protein expression of *Gpnmb*. a. Relative mRNA expression of Gpnmb in epididymal fat and liver in Gpnmb transgenic (Tg) and wild type (WT) mice. All data are presented as mean ± standard deviation (SD). n=5 **b.** Western blot of Gpnmb and calnexin in plasma membrane and cytosolic fractions in liver tissues of Gpnmb$^{-/-}$ and Gpnmb Tg mice.

Supplementary Figure 8 mRNA expression of COL1A1, MMP3, ACTA1, cell morphology and oxidative stress in LI90 cells transfected with p3xFLAG-mGpnmb. a. Relative mRNA expression of COL1A1, MMP3, ACTA1 and mGpnmb in LI90 cells transfected with p3xFLAG-mGpnmb. **b.** Immunofluorescence staining using anti-FLAG antibody in LI90 cells transfected with p3xFLAG-mGpnmb. **c.** Dihydroethidium staining and fluorescence intensity in LI90 cells transfected with p3xFLAG-mGpnmb. All data are presented as mean ± standard deviation (SD). n=3. **p<0.01 vs. LI90 cells transfected with p3xFLAG-Vector.

Supplementary Figure 9 Serum GPNMB levels in the patients with nonalcoholic fatty liver disease (NAFLD). a. Serum GPNMB levels and NAS score in the patients with NAFLD. They were classified into three groups according to the NAS score. **b.** Serum GPNMB levels and necroinflammatory grading in the patients with NAFLD. They were classified into three groups (grade 1-3) according to the degree of liver steatosis and inflammation.

Supplementary Figure 10 Schematic drawing for the function of Gpnmb. *Gpnmb* ameliorates fat accumulation and fibrosis in the liver of diet-induced obesity mice by reducing the oxidative stress. *Gpnmb* in hepatic macrophages and stellate cells interacts with calnexin and the serum soluble GPNMB is elevated in the patients with non-alcoholic

steatohepatitis.

Supplementary Figure 11 Uncropped images for the blots shown in Figure 1b, Supplementary Figure 2a, Supplementary Figure 3a, and Figure 4a.

Supplementary Figure12 Uncropped images for the blots shown in Figure 5.

Supplementary Figure 1

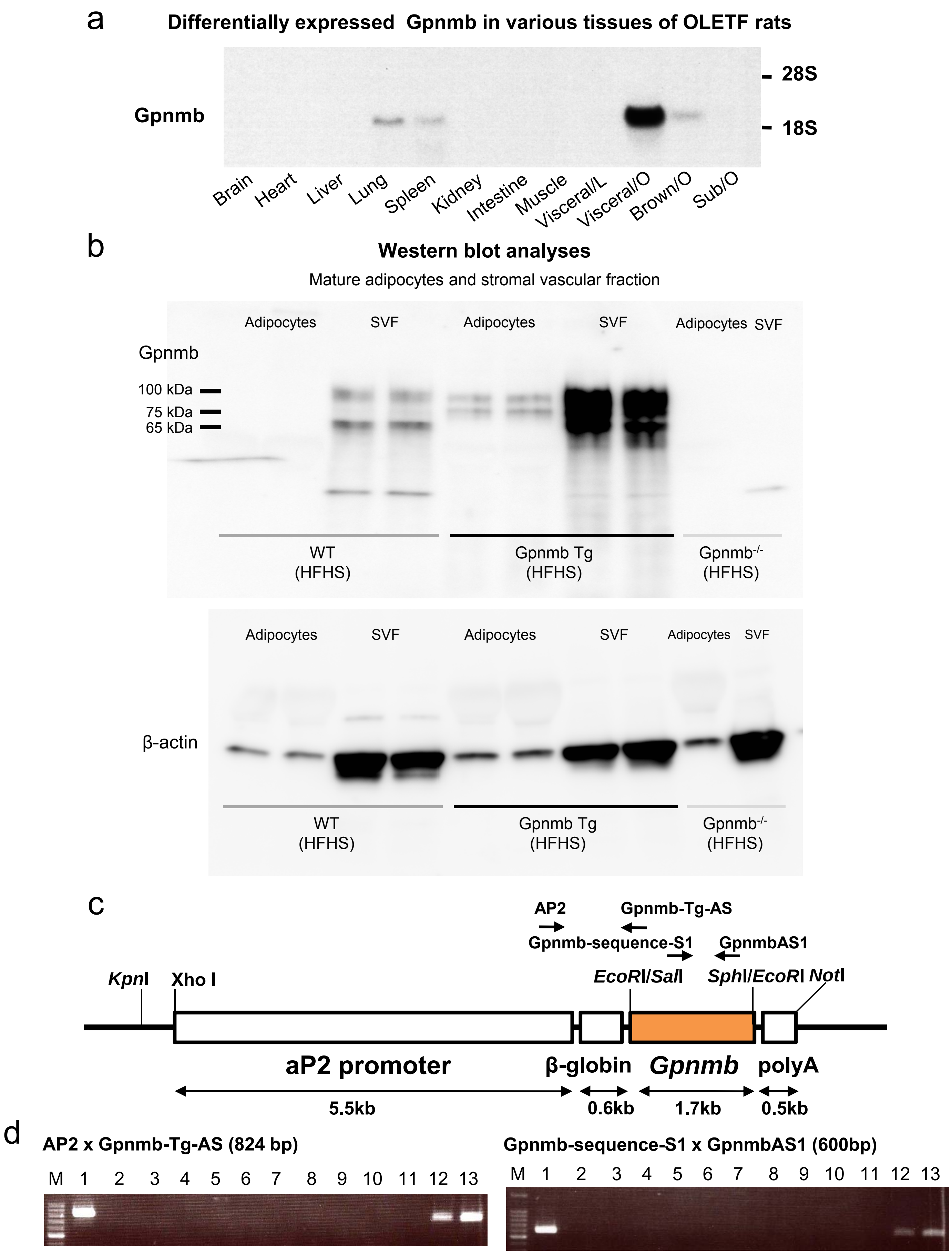

Supplementary Figure 2

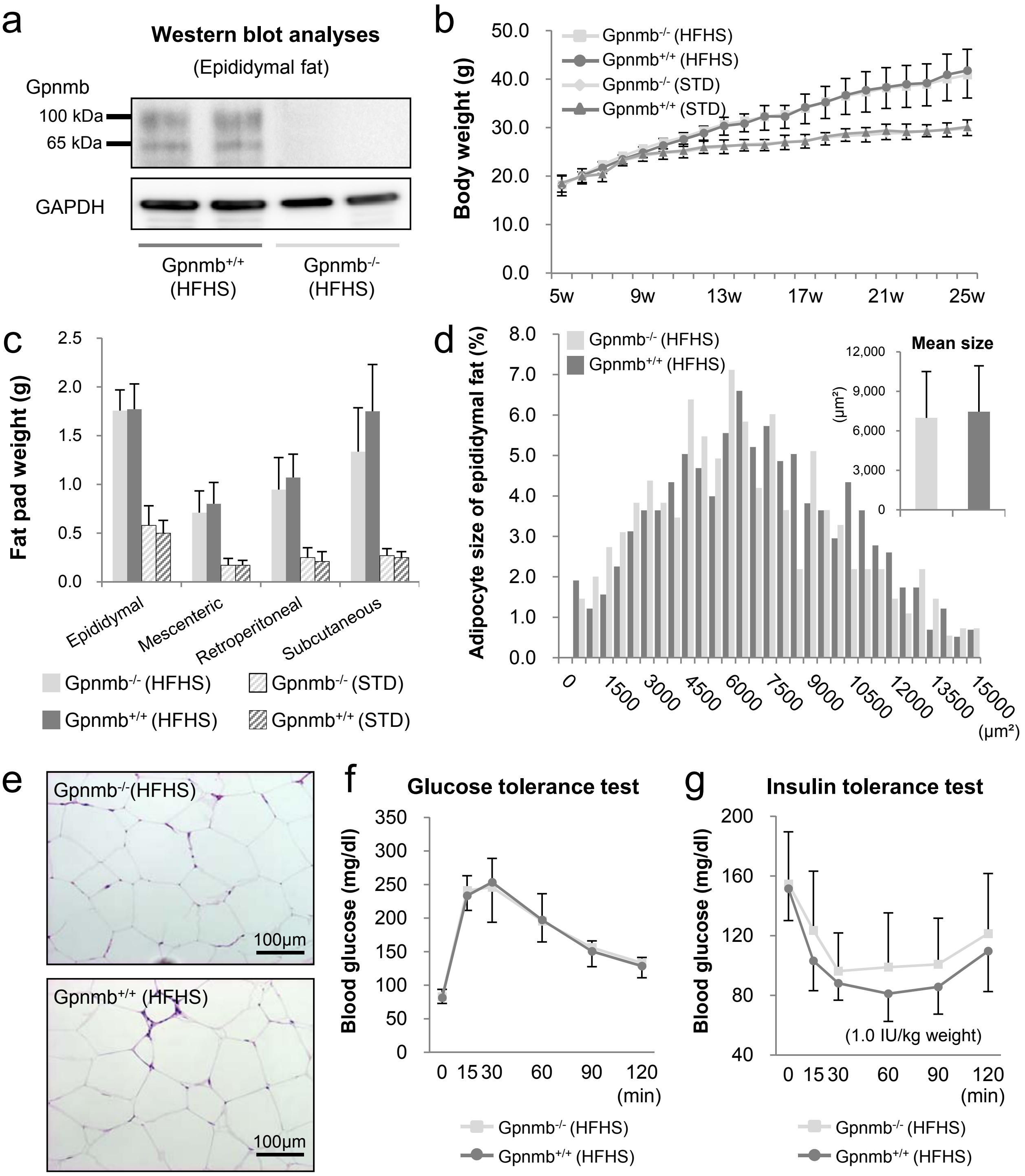

Supplementary Figure 3

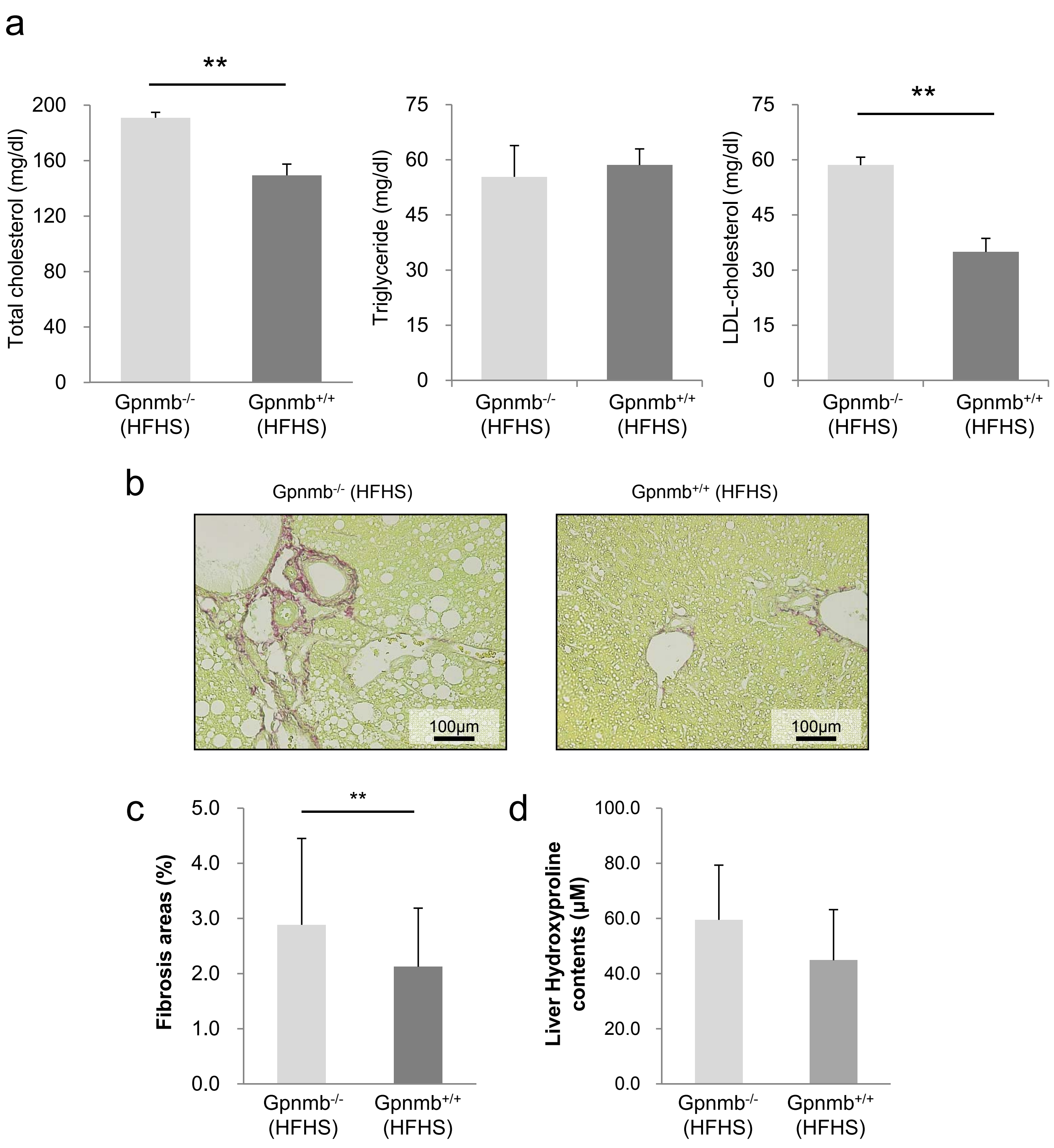

Supplementary Figure 4

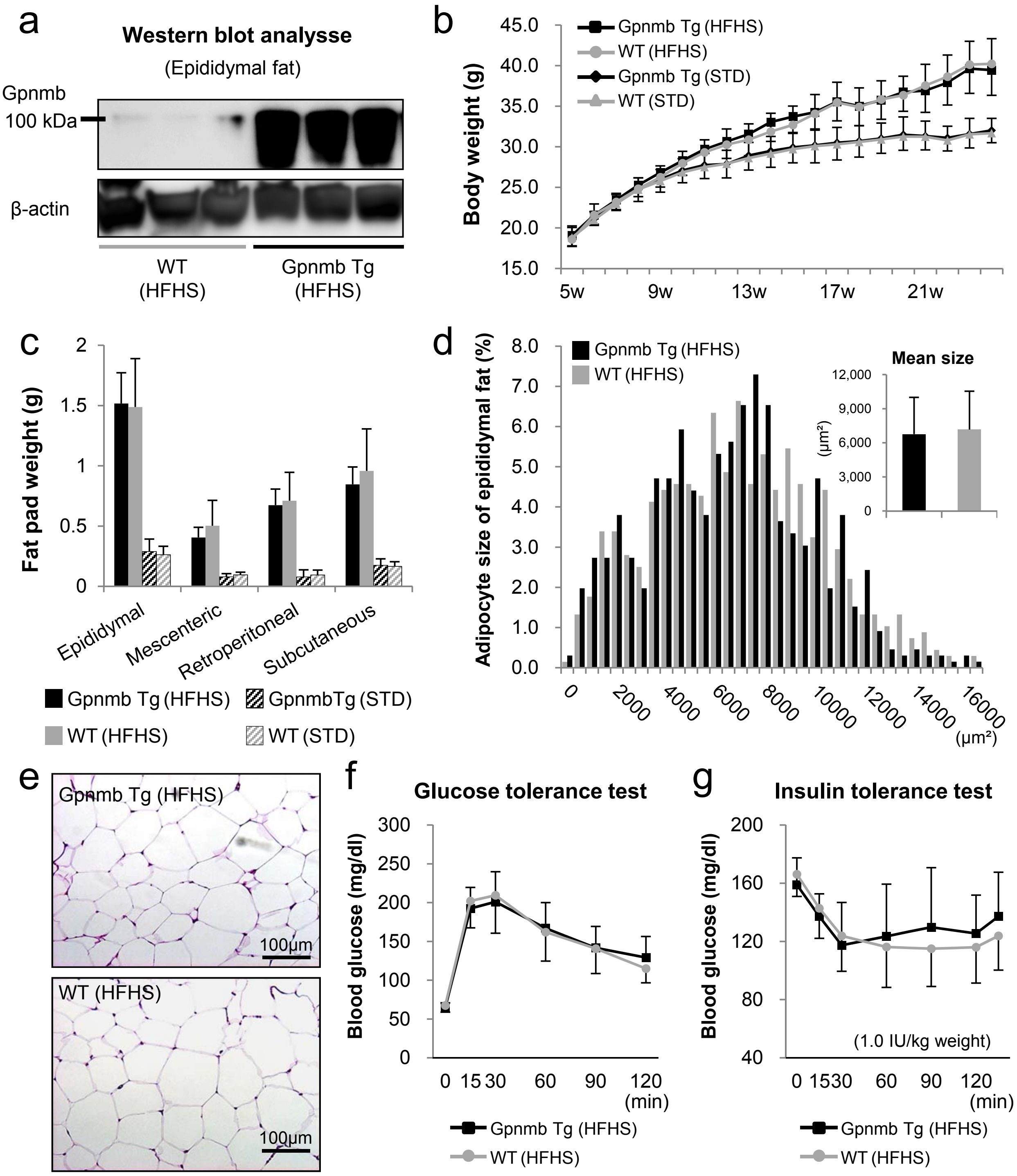

Supplementary Figure 5

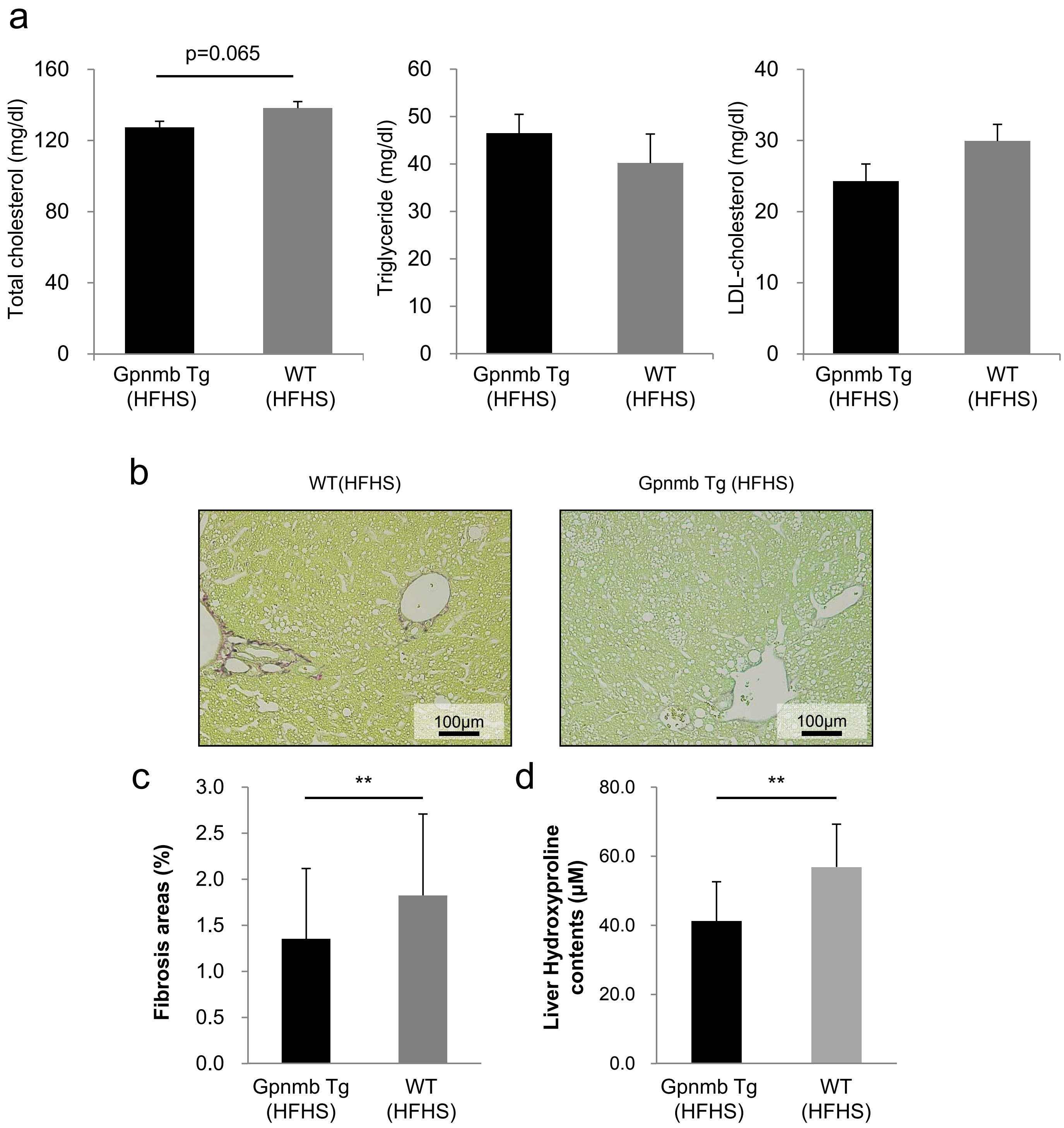

Supplementary Figure 6

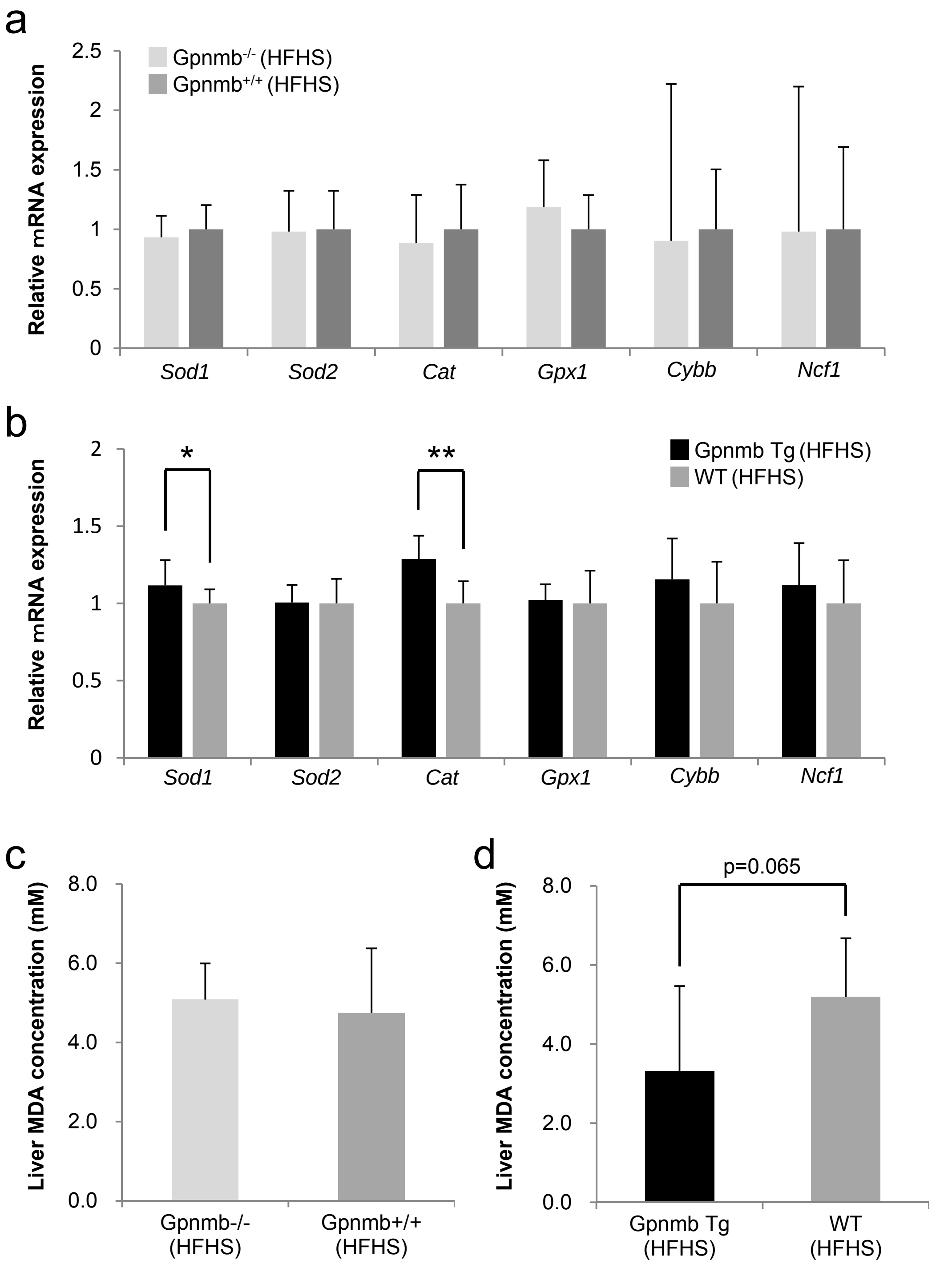

Supplementary Figure 7

a

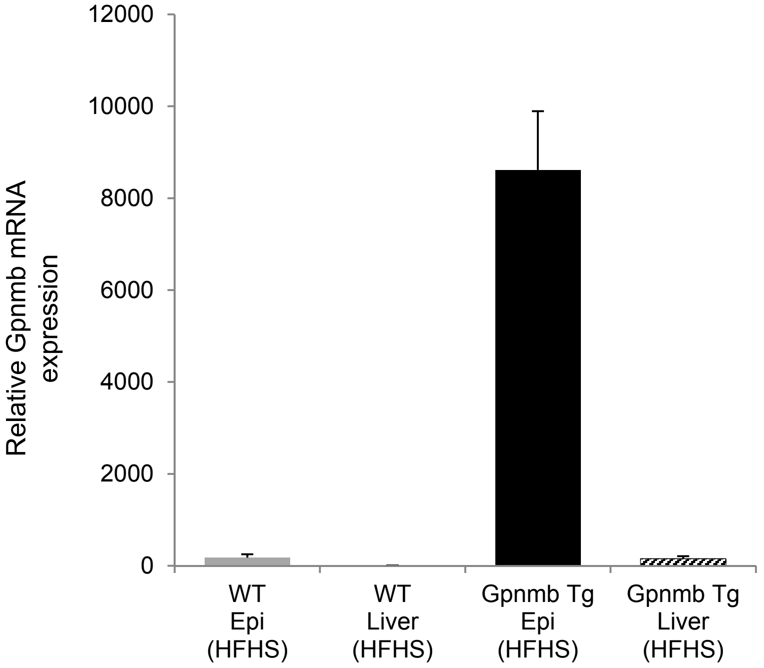

b

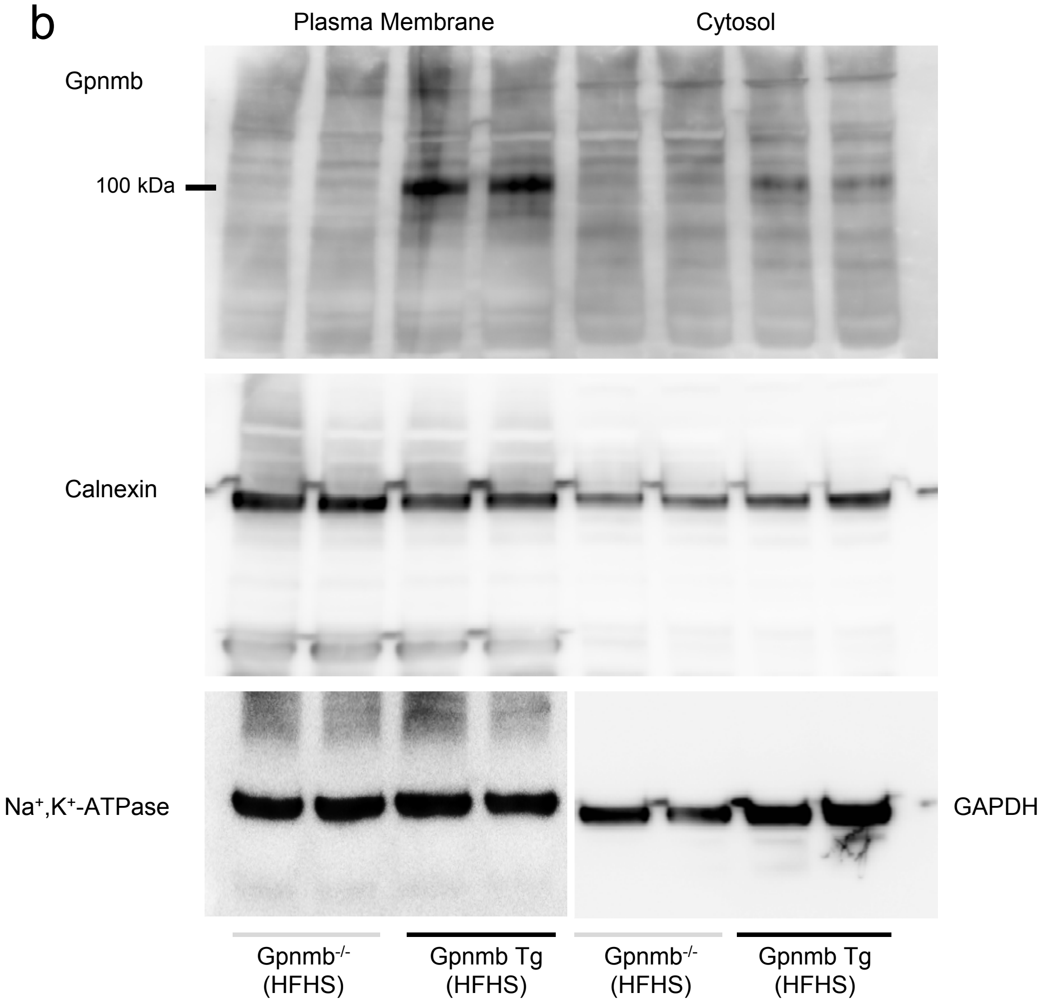

Supplementary Figure 8

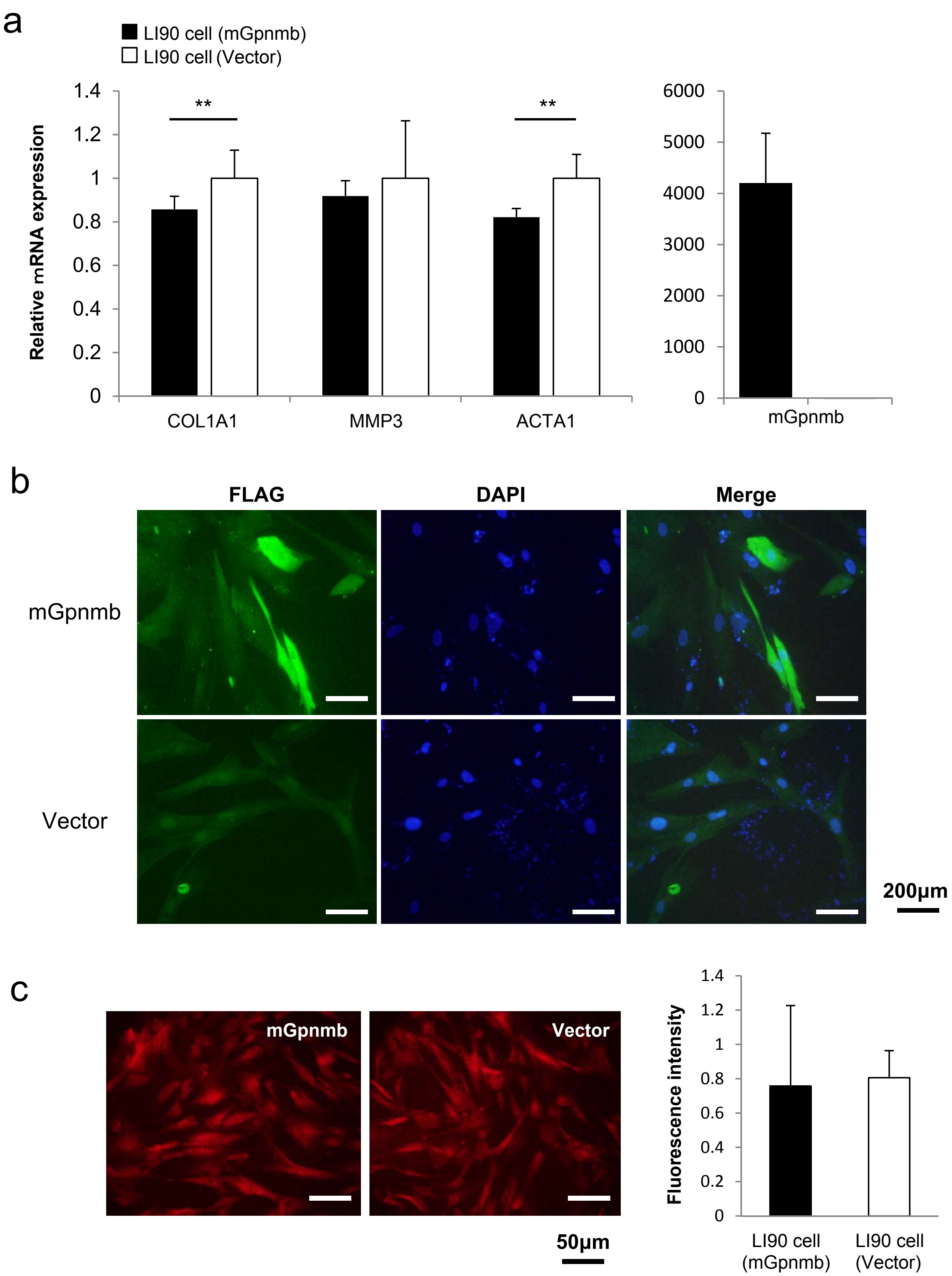

Supplementary Figure 9

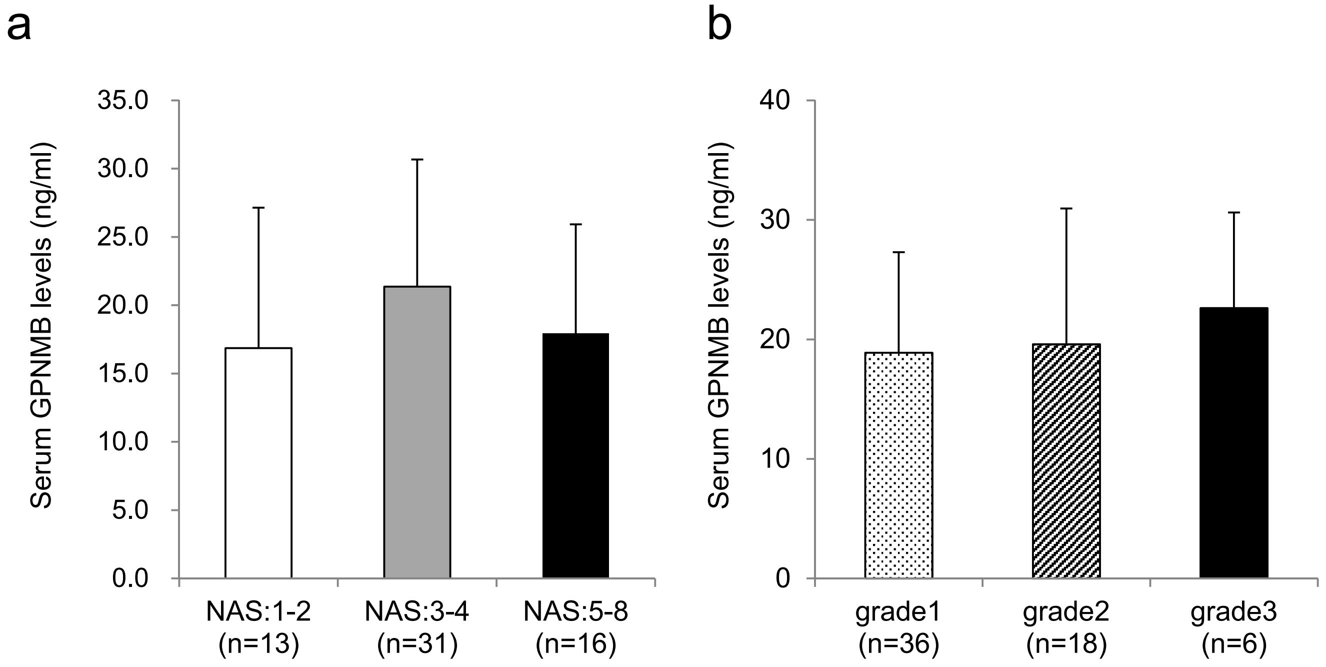

Supplementary Figure 10

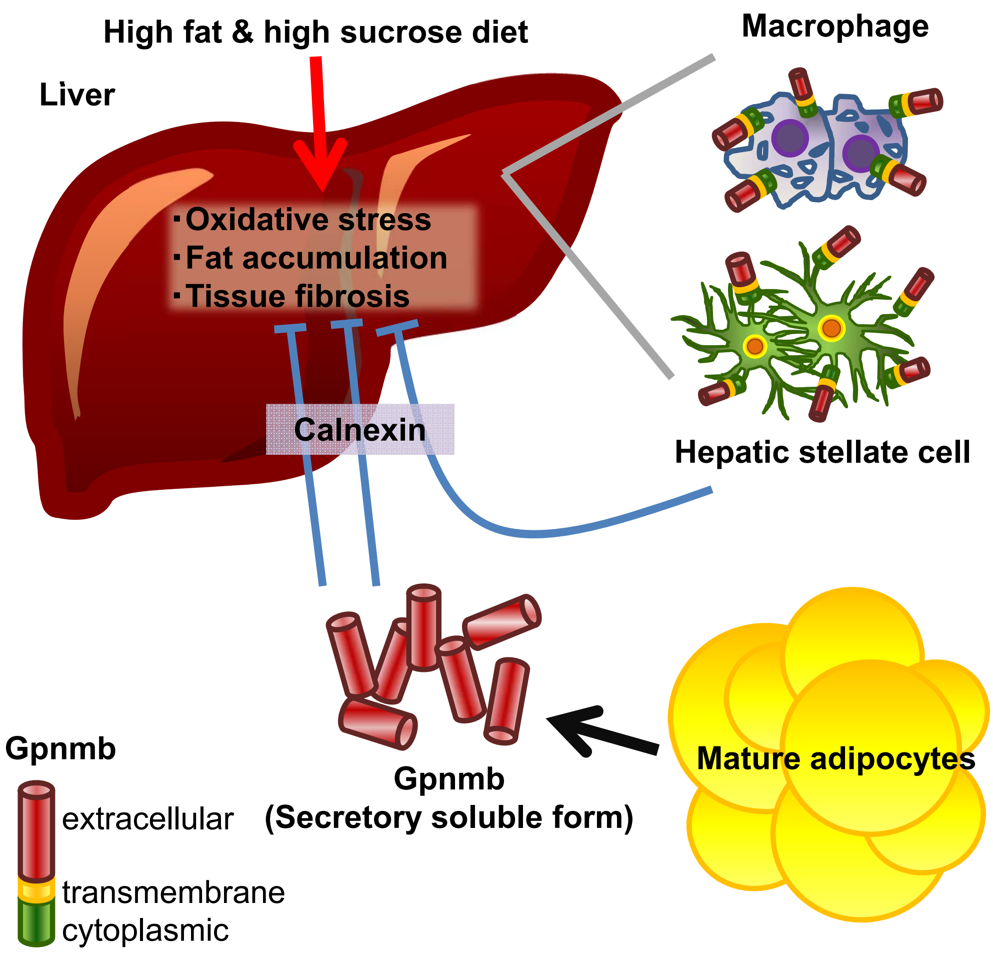

Supplementary Figure 11

a Figure 1b Original figure

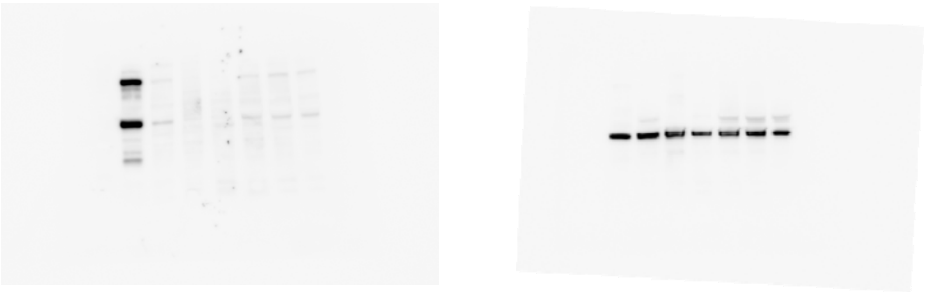

b Figure S2a Original figure

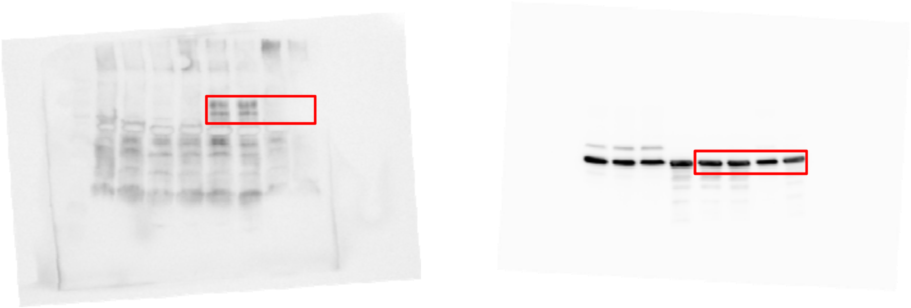

c Figure S3a Original figure

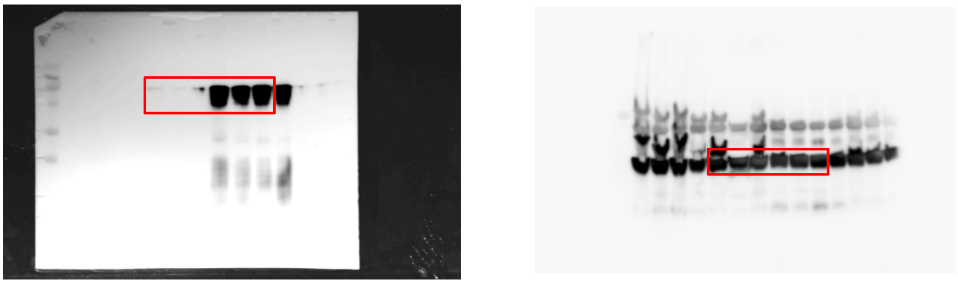

d Figure 4a Original figure

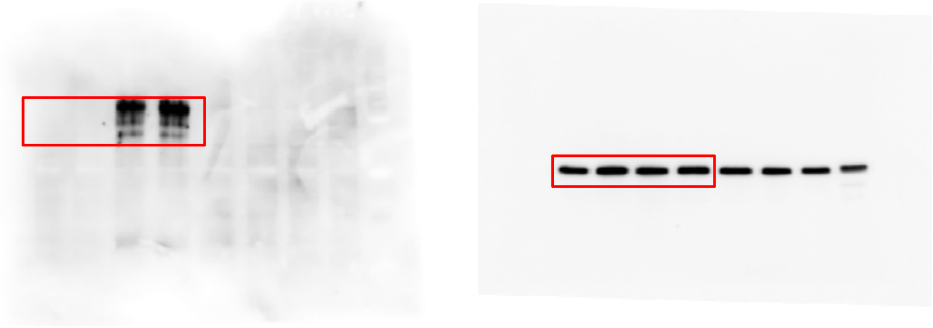

Supplementary Figure 12

a Figure 5a Original figure

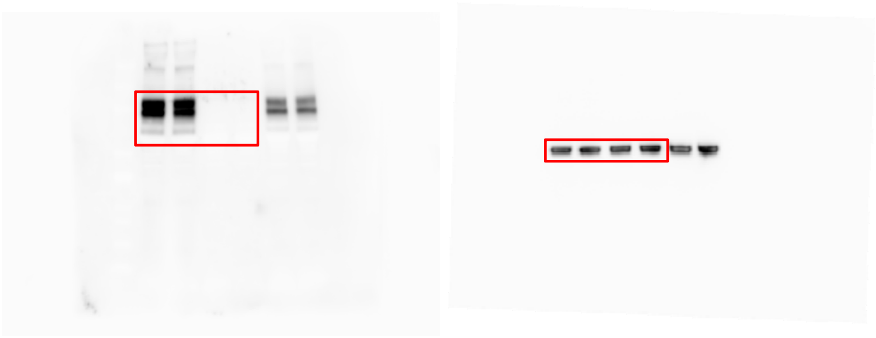

b Figure 5b Original figure

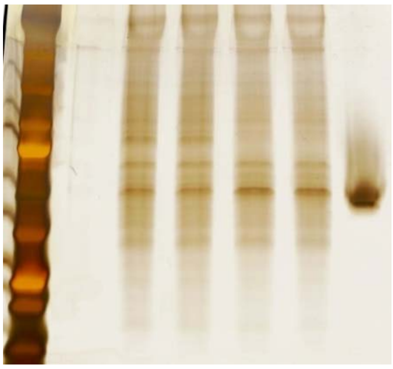

c Figure 5c Original figure

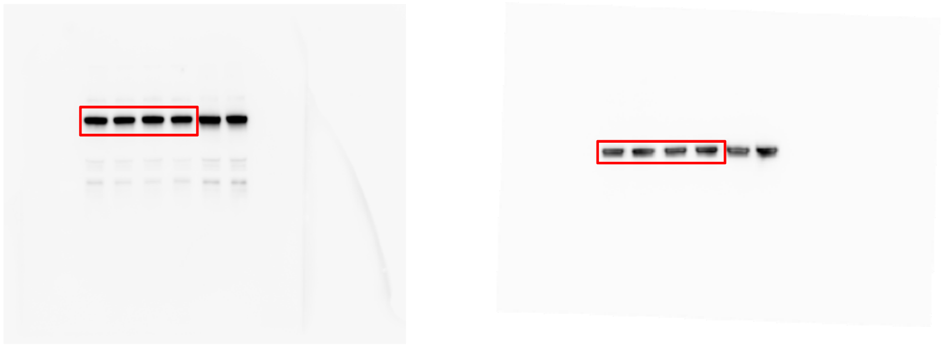

d Figure 5d Original figure

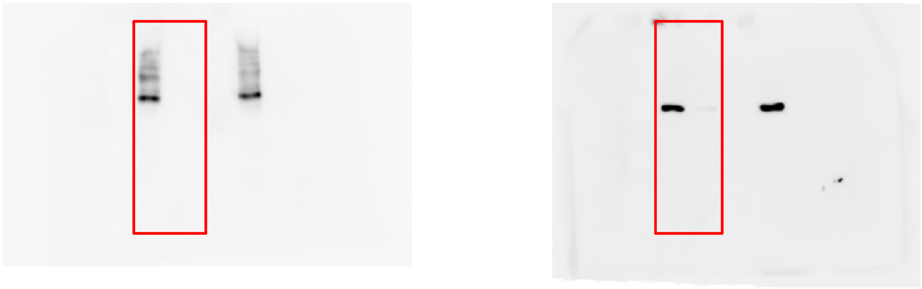

e Figure 5g Original figure

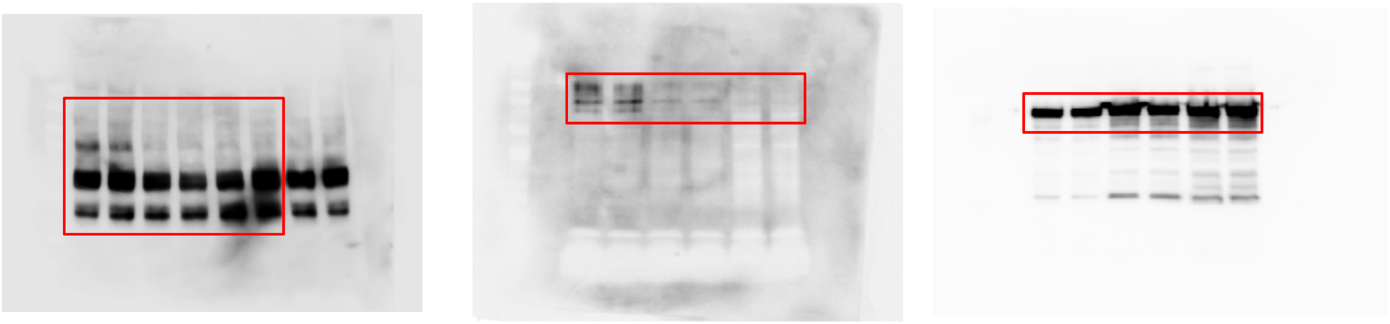

Supplement: Supplementary Information [file srep16920-s1.pdf]
